# Prediction of 90-day mortality among cancer patients with unplanned hospitalisation: a retrospective validation study of three prognostic scores

**DOI:** 10.1016/j.lanepe.2025.101317

**Published:** 2025-05-08

**Authors:** Galip Can Uyar, Oriol Mirallas, Kadriye Başkurt, Berta Martin-Cullell, Enes Yeşilbaş, Jordi Recuero-Borau, Seher Kaya, Victor Navarro Garcés, Sevgi Eryıldız Yücel, Kreina Sharela Vega Cano, Diego Gómez-Puerto, Anna Pedrola Gómez, Clara Salva de Torres, Ömür Berna Çakmak Öksüzoğlu, Sonia Serradell, Rodrigo Dienstmann, Osman Sütcüoğlu

**Affiliations:** a Medical Oncology Department, Etlik City Hospital, Ankara, Turkey; b Medical Oncology Department, Vall d’Hebron Hospital Campus and Vall d’Hebron Institute of Oncology (VHIO), Barcelona, 08035, Spain; c Medical Oncology Department, Hospital de la Santa Creu I Sant Pau, Barcelona, 08041, Spain; d Medical Oncology Department, Hospital del Mar, Barcelona, 08003, Spain; e Oncology Data Science (ODysSey) Group, Vall d’Hebron Institute of Oncology (VHIO), Barcelona, 08035, Spain

**Keywords:** Cancer, Mortality, Nutrition, Prognosis, PROMISE score

## Abstract

**Background:**

Accurate prediction of 90-day mortality in hospitalised cancer patients is critical for guiding personalised treatment decisions and optimising oncologic care. However, existing prognostic models often lack sufficient precision, particularly in distinguishing between high- and low-risk patients. In this retrospective study, we independently evaluated the prognostic performance of three scoring systems—the Prognostic Score for Hospitalised Cancer Patients (PROMISE), the Gustave Roussy Immune (GRIm) score, and the C-reactive protein–Triglyceride–Glucose Index (CTI)—in patients admitted for unplanned hospitalisations.

**Methods:**

This retrospective observational study was conducted at the Medical Oncology Clinic of Ankara Etlik City Hospital, Turkey, and included patients aged 18 years or older with a diagnosis of cancer who were hospitalised unexpectedly between February 2023 and February 2024. Laboratory data were retrieved from the institutional hospital information system. The PROMISE score was calculated using its original specification via the online tool (https://promise.vhio.net/). The GRIm score was calculated based on neutrophil-to-lymphocyte ratio (NLR), albumin, and lactate dehydrogenase (LDH). The CTI score was computed as: CTI = [0.412 × ln (C-reactive protein [CRP])] + ln [Triglyceride × Glucose/2], with a cut-off value of 4.78. A PROMISE–CTI Combined score was derived using regression-based weighting. Risk stratification was performed for all three scores using validated thresholds. Statistical analyses included Kaplan–Meier survival analysis, log-rank tests, univariable and multivariable logistic regression to assess predictors of 90-day mortality, and receiver operating characteristic (ROC) curve analysis to evaluate discriminatory performance.

**Findings:**

Among 1657 hospitalised cancer patients screened during the study period, 1109 met the inclusion criteria and were included in the analysis. PROMISE and GRIm scores were calculated for all 1109 patients, while CTI score was assessed in 333 patients with complete laboratory data. The 90-day mortality rate was 63.7% (n = 707). High PROMISE score (OR: 3.32, 95% CI: 1.40–7.86; p = 0.006) and high CTI score (OR: 2.85, 95% CI: 1.32–6.18; p = 0.008) were associated with increased 90-day mortality. Low PROMISE score (OR: 0.22, 95% CI: 0.10–0.49; p = 0.001) and low CTI score (OR: 0.35, 95% CI: 0.17–0.73; p = 0.003) were associated with reduced 90-day mortality. High GRIm score (OR: 1.83, 95% CI: 0.83–2.91; p = 0.07) and low GRIm score (OR: 0.73, 95% CI: 0.47–1.20; p = 0.08) were not significantly associated with 90-day mortality. The area under the curve (AUC) of the PROMISE–CTI Combined score was 0.884 (95% CI: 0.849–0.919; p < 0.0001). Sensitivity, specificity, positive predictive value (PPV), negative predictive value (NPV), and accuracy of the PROMISE–CTI Combined score were 92.4%, 81.1%, 85.3%, 89.6%, and 86.7%, respectively.

**Interpretation:**

The PROMISE score demonstrated strong discriminatory ability in predicting 90-day mortality among cancer patients admitted for unplanned hospitalisations. Integration of the CTI score further improved risk stratification by incorporating nutritional and inflammatory markers. The PROMISE–CTI Combined score may serve as a practical clinical tool for short-term prognostic assessment in this setting. Prospective, multicentre, randomised studies are needed to confirm the clinical utility and generalisability of the PROMISE–CTI Combined score.

**Funding:**

This study received no funding.

**Research in context**

**Evidence before this study**

We searched PubMed, Embase, and Web of Science for studies published between January 1, 2010 and November 30, 2024 using the search terms: “90-day mortality”, “unplanned hospitalisation”, “cancer patients”, “prognostic score”, “Prognostic Score for Hospitalised Cancer Patients (PROMISE) score”, “Gustave Roussy Immune (GRIm) score”, and “CRP-Triglyceride-Glucose Index (CTI) score”. No language restrictions were applied.

We included observational studies, retrospective cohorts, and model development papers assessing 90-day mortality among hospitalised adult cancer patients. Studies were excluded if they focused solely on postoperative patients, individuals admitted to intensive care units, elderly-only cohorts, or palliative populations with limited life expectancy.

The existing literature primarily comprised single-centre retrospective studies, with moderate risk of bias related to patient selection, confounding, and reporting. Notably, the PROMISE score was recently developed by Mirallas et al. in a Spanish cohort of advanced cancer patients receiving systemic treatment. However, it has not been validated outside of the original cohort. While the CTI and GRIm scores have shown prognostic relevance in cachexia and inflammation-related mortality, they have not been integrated into composite models. No study to date has externally validated the PROMISE score in a different population, nor examined its performance in combination with other nutritional or inflammatory scores. A pooled meta-analytic estimate was not feasible due to heterogeneity in study designs, endpoints, and predictive frameworks. This study is the first to validate the PROMISE score outside of Spain and to propose a novel PROMISE–CTI Combined Score for 90-day mortality risk stratification.

**Added value of this study**

To our knowledge, this is the first study to externally validate the PROMISE score outside of its original development cohort and geographical context. By applying the PROMISE score in a large and diverse real-world population, we confirmed its robust performance in predicting 90-day mortality among hospitalised cancer patients. Moreover, this is the first study to integrate the PROMISE score with the CTI score, a composite marker of nutritional and inflammatory status, thereby enhancing the score’s predictive accuracy. We also evaluated the GRIm score in this setting, allowing for a comprehensive comparison between three different prognostic systems. The novel PROMISE–CTI Combined Score proposed in this study demonstrated superior stratification ability for both low- and high-risk patients. These findings support the clinical utility of this combined model in informing risk-adapted care strategies during unplanned hospitalisations in oncology.

**Implications of all the available evidence**

Unplanned hospitalisations in oncology represent a frequent clinical challenge with substantial implications for patient outcomes, quality of care, and resource allocation. The existing evidence underscores the importance of short-term mortality prediction tools in supporting decision-making during acute care episodes. This study confirms the clinical applicability of the PROMISE score beyond its original development setting and introduces a novel composite model—PROMISE–CTI Combined Score—that incorporates inflammatory and nutritional metrics to improve prognostic accuracy. The integration of these tools into routine oncology practice may enable timely identification of low- and high-risk patients, guide early supportive care interventions, and promote risk-adapted treatment planning during hospitalisation.

To determine whether the use of this combined scoring model can lead to improved clinical outcomes, prospective, multicentre, randomised trials should be considered. Such studies are necessary to confirm the score’s generalisability and its real-world impact on decision-making, care planning, and resource utilisation in diverse oncological settings.

## Introduction

Despite significant advancements in cancer treatment, unplanned hospitalisations remain a major challenge in oncology, particularly for patients with advanced-stage disease.^1^ These hospitalisations, often driven by disease progression, treatment-related toxicities, or infections, are associated with high mortality rates, reduced quality of life, and significant financial burdens for both patients and healthcare systems.^2^, ^3^, ^4^ Early and accurate identification of high-risk patients upon hospital admission is essential for improving care quality, guiding clinical decision-making, and optimizing resources allocation.^5^

Current prognostic models for cancer patients primarily target outpatient populations and focus on parameters such as tumour burden, LDH, malnutrition, inflammatory markers, and Eastern Cooperative Oncology Group Performance Status (ECOG-PS).^6^, ^7^, ^8^, ^9^, ^10^ However, these models often fail to achieve the necessary specificity and sensitivity required to accurately predict outcomes in hospitalised patients. The rapidly evolving clinical status of these patients requires dynamic and tailored prognostic tools that adapt to the complexities of inpatient oncology care.^11^, ^12^, ^13^

The PROMISE score, developed by Mirallas and colleagues at the Vall d’Hebron Institute of Oncology (VHIO), was designed specifically for hospitalised cancer patients to address these challenges.^14^ By integrating ECOG-PS, LDH, albumin, and neutrophil count, it the PROMISE score provides a robust framework for estimating 90-day mortality. However, this model does not integrate additional indicators, such as nutritional and inflammatory markers, which are known to play pivotal roles in determining cancer prognosis.^15^ Additionally, this scoring system has been validated in Spanish cancer patients, with limited data available on its applicability and effectiveness in other populations.^14^

This study aimed to evaluate the prognostic performance of the PROMISE score in predicting 90-day mortality among hospitalised cancer patients. Additionally, it sought to independently assess the utility of nutritional and inflammatory markers, including the GRIm and CTI scores, in stratifying patients into low- and high-risk categories. By focusing on these markers, this study aimed to enhance risk stratification and support evidence-based decisions regarding treatment intensity, palliative care planning, and overall care strategies for hospitalised cancer patients.

## Methods

### Study design and patient population

This retrospective study was conducted at the Medical Oncology Clinic of Ankara Etlik City Hospital, a major cancer reference centre in Turkey. Data were collected over a one-year period, from February 2023 to February 2024. The study was conducted in accordance with the Declaration of Helsinki and Good Clinical Practice (GCP) guidelines. Ethical approval was obtained from the Ankara Etlik City Hospital Scientific Research and Evaluation Ethics Committee (Approval number: 2024-965).

### Patient selection

**Fig. 1:**
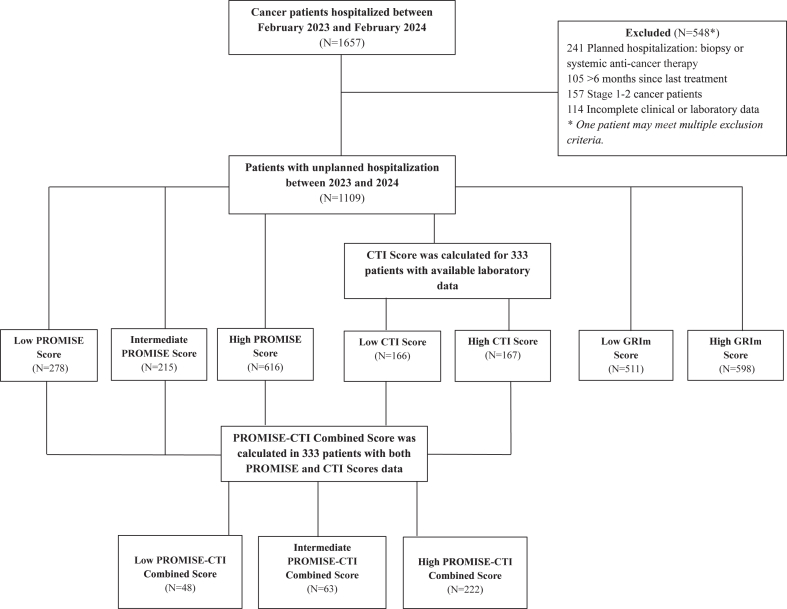
**Flowchart of patient selection and inclusion.** A total of 1657 hospital admissions were screened. After excluding patients with planned admissions, missing laboratory data, or stage I–II cancers, 1109 patients were included in the final analysis. PROMISE and GRIm scores were calculated for all eligible patients, while the CTI score was evaluated in a subset of 333 patients with sufficient laboratory data.

Patients aged 18 years or older with stage 3 or 4 cancer who were receiving active anti-cancer therapy (e.g., chemotherapy, targeted therapy, or immunotherapy) or had completed such treatment within the previous six months were included in the study (n = 1109). Only unplanned hospitalisations were included. Patients with stage 1 or 2 cancer, planned hospitalisations for anti-cancer therapy or interventional procedures (e.g., biopsy), and those excluded due to incomplete clinical or laboratory data (n = 548, 33.1%) were not included in the study. Recurrent admissions meeting the inclusion criteria were included in the analysis. The patient selection process and risk stratification are illustrated in Fig. 1.

### Reasons for hospitalisation

Hospitalisations were categorized into 11 distinct groups based on the primary clinical complaints at admission, following the International Classification of Diseases, 11th Revision (ICD-11) framework provided by the World Health Organization (WHO).^16^^,^^17^ These categories included palliative symptoms, gastrointestinal system (GIS) issues, infections, haematological issues, respiratory problems, electrolyte imbalances, neurological symptoms, nephrological issues, oedema, allergic reactions, or circulatory disorders.^17^

### Data collection

Data for the study were retrospectively collected by the researchers from patient files and the hospital information system of the Ankara Etlik City Hospital Medical Oncology Clinic. These records included demographic information, body mass index (BMI),^18^ ECOG-PS,^19^ smoking history, reasons for hospitalisation, modified Charlson Comorbidity Index (mCCI),^20^ cancer types, diabetes or dyslipidaemia status, the number of systemic therapy lines categorized for both metastatic and non-metastatic patients within the study population, and laboratory findings. Laboratory and clinical parameters collected within the first 24 h of hospitalisation were included for subsequent analyses.

### Prognostic scoring systems

Three prognostic scoring systems—PROMISE, GRIm, and CTI scores—were employed to assess risk and predict 90-day mortality.

The PROMISE Score was calculated using the “PROMISE Score Calculation Tool” (https://promise.vhio.net/). This score incorporated ECOG-PS, LDH, serum albumin levels, neutrophil counts and oncologic treatment response. Patients were categorized into three risk groups: low risk (<27.33%), medium risk (27.33–53.04%), and high risk (>53.04%). The PROMISE score aims to stratify hospitalised cancer patients into low- and high-risk groups, facilitating risk assessment and clinical decision-making.^14^

The GRIm Score was derived from serum albumin levels (<35 g/dL), LDH levels (>225 UI/L), and the NLR > 6. Patients with a GRIm score of 0–1 were considered low risk, whereas those scoring 2–3 were categorized as high risk. This score provided a quick and reliable assessment of inflammation and nutritional status.^21^^,^^22^

The CTI Score was calculated using a combination of C-reactive protein (CRP) levels and the TyG index (Triglyceride-Glucose Index). The TyG index was determined using the formula: TyG = ln (Triglyceride [mg/dL] × Fasting Glucose [mg/dL])/2. The CTI score was then computed as: CTI = [0.412 × ln (CRP)] + TyG. Based on previous research by Ruan et al., a CTI threshold of 4.78 was used to classify patients into low-risk (<4.78) and high-risk (≥4.78) groups. The CTI score has been shown to be particularly useful in assessing the combined effect of nutritional and inflammatory markers on patient prognosis and might predict cancer cachexia.^23^^,^^24^

To construct the PROMISE-CTI Combined score, multivariable logistic regression analysis was performed to identify independent predictors of 90-day mortality. Regression coefficients (β) obtained from this model were used to formulate the combined score as follows:

$$\text{PROMISE}-\text{CTI}\;\text{Combined}\;\text{Score}=\left(\beta 1\times \text{PROMISE}\;\text{Score}\right)\;+\;\left(\beta 2\times \text{CTI}\;\text{Score}\right)$$

The analysis yielded β_1_ = 0.045 for the PROMISE score and β_2_ = 1.112 for the CTI score. Based on this formula, ROC curve analysis was performed to determine the optimal cutoff points for risk classification. Patients were stratified into low, intermediate, and high-risk groups, with the following thresholds: low risk: ≤6.5, intermediate risk: 6.5–8.0, and high risk: ≥8.0. The prognostic performance of the PROMISE-CTI Combined score in predicting 90-day mortality was assessed by calculating sensitivity, specificity, positive predictive value (PPV), negative predictive value (NPV), and accuracy. Furthermore, its ability to discriminate between high-risk and low-risk patients was evaluated, along with its independent predictive power for 90-day mortality, as demonstrated by the PROMISE and CTI scores.

### Outcome definition

Ninety-day mortality, defined as death occurring within 90 days of hospital admission, was chosen as a clinically relevant timeframe to evaluate the prognostic accuracy and utility of the scoring systems in unplanned hospitalisations of cancer patients.

### Statistical analysis

All statistical analyses were conducted using SPSS version 25.0 (IBM Corporation, Armonk, NY, USA) and R version 4.2.2 (R Foundation for Statistical Computing, Vienna, Austria). Normality of continuous variables was assessed using the Kolmogorov–Smirnov and Shapiro–Wilk tests, as well as visual inspection of histograms and skewness statistics. As none of the continuous variables were normally distributed, all continuous data were summarised using medians with interquartile ranges (IQRs) and compared between groups using the Mann–Whitney U test. Categorical variables were expressed as frequencies and percentages and compared using the Pearson Chi-square test. Correlation analysis between PROMISE, GRIm, and CTI scores was performed using Spearman’s rank correlation coefficient. A p-value <0.05 was considered statistically significant for all correlation analyses. Survival analysis was conducted using Kaplan–Meier curves, with differences between groups assessed by the log-rank test. Independent prognostic factors for 90-day mortality were identified through logistic regression analysis, with odds ratios (OR) and 95% confidence intervals (CIs) reported. Variables with p < 0.10 in univariate analysis were included in the multivariable model, and statistical significance was set at p < 0.05. Effect size analysis was performed using Cohen’s d, with values classified as small (d < 0.2), medium (0.2 ≤ d < 0.5), large (0.5 ≤ d < 0.8), and very large (d ≥ 0.8). Number Needed to Treat (NNT) analysis was performed for both low-risk and high-risk categories of the PROMISE and CTI scores to estimate the number of patients required in the low-risk group to prevent one mortality event.

A multivariable logistic regression analysis was performed to identify independent predictors of 90-day mortality, and the resulting regression coefficients (β) were used to develop the PROMISE-CTI Combined Score. Discriminative ability was evaluated using Receiver Operating Characteristic (ROC) analysis, with Area Under the Curve (AUC) values and 95% CIs reported. Previously validated cutoff values were applied for the PROMISE and CTI scores, while the optimal threshold for the PROMISE-CTI Combined Score was identified using the Youden Index. Sensitivity, specificity, positive predictive value (PPV), negative predictive value (NPV), and overall accuracy were calculated for each scoring method. Model fit was assessed via the Hosmer–Lemeshow test (p > 0.05 indicating a good fit), and Nagelkerke R^2^ was used to estimate predictive strength. To improve model calibration, a logit transformation was applied to predicted probabilities, followed by linear regression analysis, with model fit assessed using R^2^ and ANOVA. The Brier score was used to quantify calibration quality (values ≤ 0.25 considered acceptable), and a calibration plot was generated to compare observed and predicted mortality rates.

### Role of the funding source

No funding was received for this study.

## Results

A total of 1657 hospital admissions occurred during the study period. Patients with missing laboratory data, those with planned hospitalisations, and those with stage 1 or 2 cancer were excluded. Consequently, 1109 patients who met the inclusion criteria were included in the analysis. Among them, PROMISE and GRIm scores were calculated for all 1109 patients, while a subset of 333 patients with sufficient laboratory data was included for CTI score evaluation. The median age of the study population was 64 years (IQR: 56–71), and 595 patients (53.7%) were younger than 65. The population included 41.4% (n = 459) female patients. ECOG-PS assessment revealed that 386 patients (34.8%) had an ECOG-PS of 0–1, while 723 patients (65.2%) had an ECOG-PS of ≥2. The median BMI of the study population was 22.7 kg/m^2^ (IQR: 19.5–26.4), and the median weight was 67.5 kg (IQR: 59.1–76.8). According to the PROMISE score, 278 patients (25.1%) were categorized as low risk, 215 patients (19.4%) as intermediate risk, and 616 patients (55.5%) as high risk. The GRIm score classified 511 patients (46.0%) as low risk and 598 patients (54.0%) as high risk. Among the 333 patients evaluated using the CTI score, 166 patients (49.8%) were categorized as low risk and 167 patients (50.2%) as high risk. Detailed clinical and demographic data are shown in Table 1. No significant differences in baseline clinical characteristics were observed between patients with and without available CTI scores. This suggests that missing CTI score data are unlikely to introduce selection bias (Supplementary Table S1).

**Table 1:** Baseline demographic, clinical, and risk stratification characteristics.

Parameters	All patients, n = 1109 (%) or (IQR)	PROMISE score, n = 1109 (%) or (IQR)			GRIm score, n = 1109 (%) or (IQR)		CTI score, n = 333 (%) or (IQR)	
		Low risk, n = 278 (25.1%)	Intermediate risk, n = 215 (19.4%)	High risk, n = 616 (55.5%)	Low risk, n = 510 (46.0%)	High risk, n = 599 (54.0%)	Low risk, n = 166 (49.8%)	High risk, n = 167 (50.2%)
**Age**								
<65	595 (53.7)	182 (16.4)	130 (11.7)	283 (25.4)	304 (27.4)	291 (26.2)	102 (30.6)	87 (26.1)
≥65	514 (46.3)	96 (8.7)	85 (7.7)	333 (30.1)	206 (18.6)	308 (27.8)	64 (19.2)	80 (24.1)
**Gender**								
Female	459 (41.4)	143 (12.9)	107 (9.6)	209 (18.8)	239 (21.6)	220 (19.9)	85 (25.5)	66 (19.8)
Male	650 (58.6)	135 (12.2)	108 (9.8)	407 (36.7)	271 (24.4)	379 (34.1)	81 (24.3)	101 (30.4)
**ECOG-PS**								
0–1	386 (34.8)	244 (22.1)	109 (9.9)	33 (2.9)	320 (28.9)	66 (5.9)	112 (33.6)	24 (7.3)
≥2	723 (65.2)	34 (3.0)	106 (9.5)	583 (52.6)	190 (17.1)	533 (48.1)	54 (16.2)	143 (42.9)
**Diagnosis**								
GIS	350 (31.6)	84 (7.5)	76 (6.8)	190 (17.1)	147 (13.2)	203 (18.3)	45 (13.5)	50 (15.0)
Lung	283 (25.5)	42 (3.8)	56 (5.0)	185 (16.6)	114 (10.2)	169 (15.2)	32 (9.6)	30 (9.0)
Breast	92 (8.3)	36 (3.2)	14 (1.2)	42 (3.7)	44 (3.9)	48 (4.3)	26 (7.8)	28 (8.4)
Genitourinary	89 (8.0)	31 (2.8)	10 (0.9)	48 (4.3)	47 (4.2)	42 (3.7)	12 (3.6)	17 (5.1)
Gyn-oncology	84 (7.6)	32 (2.9)	23 (2.1)	29 (2.6)	49 (4.4)	35 (3.1)	15 (4.5)	14 (4.2)
Head-Neck	57 (5.2)	16 (1.4)	8 (0.7)	33 (2.9)	36 (3.2)	21 (1.9)	5 (1.5)	7 (2.1)
Brain	44 (3.9)	3 (0.3)	12 (1.1)	29 (2.6)	14 (1.2)	30 (2.7)	8 (2.4)	8 (2.4)
Others	110 (9.9)	34 (3.0)	16 (1.4)	60 (5.4)	59 (5.3)	51 (4.5)	23 (6.9)	13 (3.9)
**Treatment response**								
PD	729 (65.7)	29 (2.6)	116 (10.5)	584 (52.7)	197 (17.7)	532 (47.9)	46 (13.8)	132 (39.6)
Non-PD	380 (34.3)	249 (22.4)	99 (8.9)	32 (2.8)	313 (28.3)	67 (6.1)	120 (36.0)	35 (10.5)
**Smoking status**								
Non-smoker	262 (23.6)	112 (10.1)	59 (5.3)	91 (8.2)	159 (14.3)	103 (9.3)	65 (19.5)	26 (7.8)
Former smoker	733 (66.1)	137 (12.3)	132 (11.9)	464 (41.8)	293 (26.4)	440 (39.7)	82 (24.6)	119 (5.7)
Active smoker	114 (10.3)	29 (2.6)	24 (2.1)	61 (5.5)	58 (5.3)	56 (5.0)	19 (5.7)	22 (30.0)
**Dyslipidemia**								
Absent	912 (82.2)	253 (22.8)	172 (15.5)	487 (43.9)	444 (40.0)	468 (42.2)	139 (41.7)	116 (34.8)
Present	197 (17.8)	25 (2.3)	43 (3.9)	129 (11.6)	66 (6.0)	131 (11.8)	27 (8.1)	51 (15.3)
**Diabetes diagnosis**								
Absent	806 (72.7)	229 (20.6)	159 (14.4)	418 (37.6)	399 (36.0)	407 (36.7)	134 (40.2)	103 (30.9)
Present	303 (27.3)	49 (4.4)	56 (5.0)	198 (17.9)	111 (10.0)	192 (17.3)	32 (9.6)	64 (19.2)
**Line of systemic therapy**								
1–2	988 (89.1)	257 (23.2)	196 (17.7)	535 (48.2)	463 (41.8)	525 (47.4)	149 (44.7)	139 (41.7)
≥3	121 (10.9)	21 (1.9)	19 (1.7)	81 (7.3)	47 (4.2)	74 (6.6)	17 (5.1)	28 (8.4)
**BMI (kg/m^2^)**								
<22.5	411 (37.1)	103 (9.4)	79 (7.1)	229 (20.6)	189 (17.1)	222 (20.0)	54 (16.2)	63 (18.9)
≥22.5	698 (62.9)	175 (15.7)	136 (12.3)	387 (34.9)	321 (28.9)	377 (34.0)	112 (33.6)	104 (31.2)
**Reasons for hospitalization**								
Palliative	249 (22.5)	78 (7.0)	55 (5.0)	116 (10.5)	147 (13.3)	102 (9.2)	43 (12.9)	30 (9.0)
GIS	203 (18.3)	53 (4.8)	46 (4.1)	104 (9.4)	98 (8.8)	105 (9.5)	32 (9.6)	28 (8.4)
Infection	159 (14.3)	28 (2.5)	22 (2.0)	109 (9.8)	58 (5.2)	101 (9.1)	12 (3.6)	35 (10.5)
Hematological	137 (12.4)	48 (4.3)	27 (2.4)	62 (5.6)	71 (6.4)	66 (6.0)	22 (6.6)	21 (6.3)
Respiratory	124 (11.2)	9 (0.8)	17 (1.5)	98 (8.8)	28 (2.5)	96 (8.7)	9 (2.7)	17 (5.1)
Electrolyte imbalance	88 (7.9)	31 (2.8)	16 (1.4)	41 (3.7)	48 (4.3)	40 (3.6)	23 (6.9)	11 (3.3)
Neurological	51 (4.6)	7 (0.6)	7 (0.6)	37 (3.3)	14 (1.3)	37 (3.3)	5 (1.5)	11 (3.3)
Nephrological	48 (4.4)	13 (1.2)	10 (0.9)	25 (2.3)	23 (2.1)	25 (2.3)	8 (2.4)	8 (2.4)
Edema	33 (3.0)	6 (0.5)	10 (0.9)	17 (1.5)	14 (1.3)	19 (1.7)	8 (2.4)	4 (1.2)
Allergic	9 (0.8)	4 (0.4)	4 (0.4)	1 (0.1)	7 (0.6)	2 (0.2)	2 (0.6)	1 (0.3)
Circulatory disorders	8 (0.7)	1 (0.1)	1 (0.1)	6 (0.5)	2 (0.2)	6 (0.5)	2 (0.6)	1 (0.3)
Age, median (years)	64 (56–71)	61 (52–66)	60 (53–68)	65 (57–71)	63 (54–69)	64 (56–71)	61 (53–67)	64 (56–72)
LDH, median (UI/L)	273 (184–368)	200 (166–227)	231 (183–278)	363 (206–594)	204 (168–239)	358 (193–577)	214 (168–258)	333 (170–577)
CRP, median (mg/L)	49.5 (15.3–94.7)	10.5 (3.7–21.9)	41.7 (15.1–89.2)	75.7 (29.3–128.3)	12.3 (3.9–41.0)	84.2 (36.4–135.4)	10.7 (3.5–23.3)	94.3 (36.6–122.0)
Albumin, median (g/dl)	33.7 (29.3–38.1)	38.9 (35.8–41.0)	36.0 (31.8–39.8)	29.7 (26.6–33.1)	37.8 (34.3–40.5)	30.2 (26.7–33.0)	38.2 (34.8–41.7)	30.5 (26.6–33.8)
PMN, median (/mm^3^)	7.4 (4.6–9.4)	7.1 (5.1–7.8)	7.58 (5.6–10.3)	8.1 (5.7–14.6)	7.2 (5.9–8.3)	8.3 (6.0–15.1)	7.2 (5.6–8.7)	8.4 (6.2–15.1)
mCCI, median	9 (8–10)	8 (7–9)	9 (8–10)	9 (9–10)	9 (8–10)	9 (9–10)	8 (7–9)	9 (8–10)
BMI, median (kg/m^2^)	22.7 (19.5–26.4)	23.1 (19.9–26.3)	22.9 (19.7–26.6)	22.5 (19.3–26.2)	23.1 (19.8–26.2)	23.0 (19.8–26.0)	23.2 (20.1–26.4)	22.3 (19.3–25.6)

BMI: Body Mass Index, CRP: C-Reactive Protein, CTI score: CRP-Triglyceride-Glucose Index, ECOG-PS: Eastern Cooperative Oncology Group-Performance Status, GIS: Gastrointestinal System, GRIm score: Gustave Roussy Immune Score, IQR: Interquartile Range, Non-PD: Non-Progressive Disease, LDH: Lactate Dehydrogenase, mCCI: Modified Charlson Comorbidity Index, PD: Progressive Disease, PMN: Polymorphonuclear Cell Count, PROMISE score: Prognostic Score for Hospitalized Cancer Patients.

**Fig. 2:**
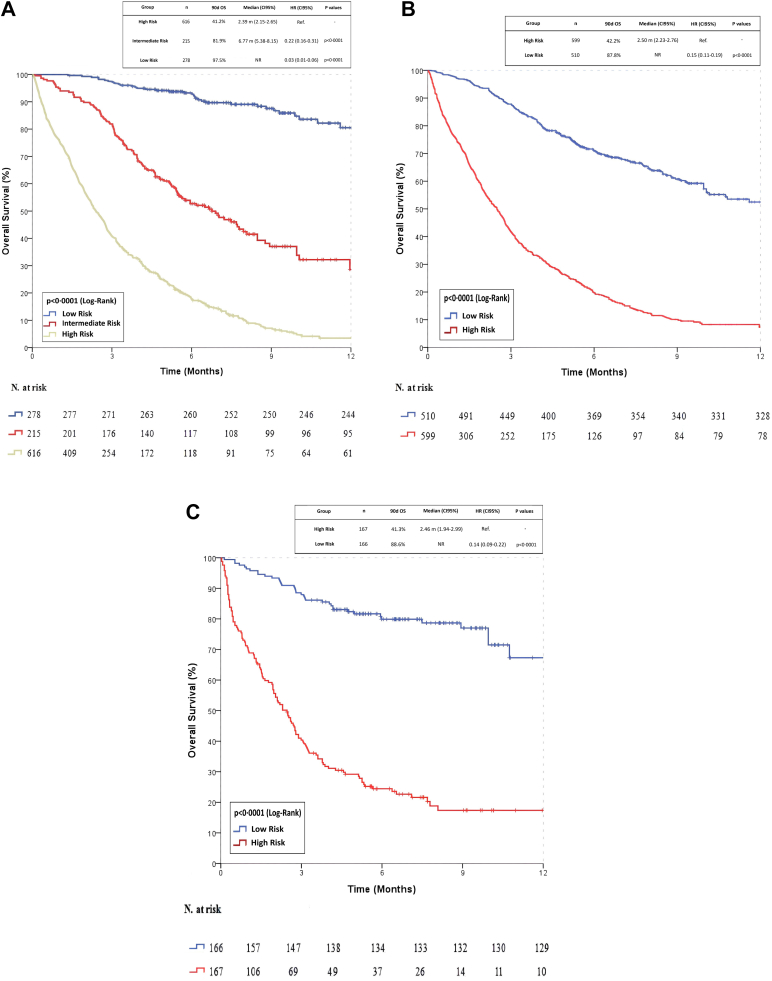
**Kaplan–Meier survival curves for risk stratification using PROMISE, GRIm, and CTI scores.** (A) PROMISE score-based survival analysis (n = 1109), (B) GRIm score-based survival analysis (n = 1109), (C) CTI score-based survival analysis (n = 333). The log-rank test was used to compare survival differences between risk groups.

The overall 90-day mortality rate was 63.7% (n = 707). For the PROMISE score, 271 patients (97.5%) in the low-risk group survived, with a 90-day mortality rate of 2.5% (n = 7). In the intermediate-risk group, 176 patients (81.9%) survived, and the 90-day mortality rate was 18.1% (n = 39). In the high-risk group, 254 patients (41.2%) survived, with a 90-day mortality rate of 58.8% (n = 362) (p < 0.0001). According to the PROMISE score, the median overall survival (OS) for the entire cohort was 4.78 months (95% CI: 4.29–5.28). For the GRIm score, the 90-day survival rate was 87.8% (n = 449) for low-risk patients and 42.2% (n = 252) for high-risk patients (p < 0.0001). The estimated median OS for the entire cohort was 4.63 months (95% CI: 4.14–5.12). Similarly, in CTI score assessments, low-risk patients demonstrated an 88.6% survival rate (n = 147), while high-risk patients had a 41.3% survival rate (n = 69) (p < 0.0001). Median OS for the overall cohort was 7.49 months (95% CI: 4.92–10.06). Kaplan–Meier survival analyses for the PROMISE, GRIm, and CTI scores are presented in Fig. 2.

**Fig. 3:**
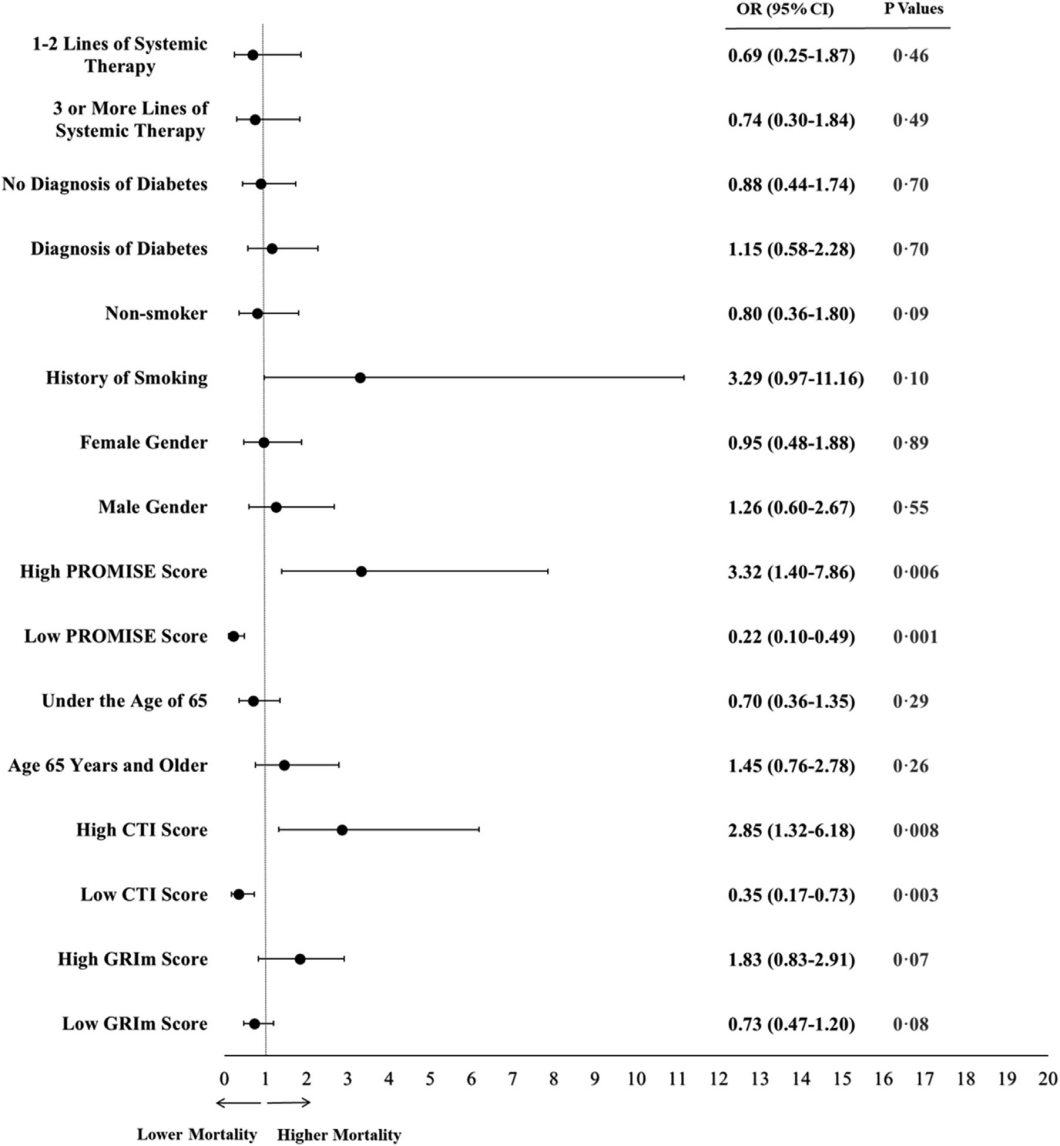
**Independent prognostic and protective factors for 90-day mortality: multivariable analysis results.** Forest plot showing odds ratios (ORs) and 95% confidence intervals (CIs) for each variable.

Univariable analysis identified several factors significantly associated with 90-day mortality, including PROMISE Score (OR: 6.93, 95% CI: 5.32–9.25; p < 0.0001), GRIm Score (OR: 9.88, 95% CI: 7.28–13.58; p < 0.0001), CTI Score (OR: 10.98, 95% CI: 6.35–19.87; p < 0.0001), age (OR: 1.67, 95% CI: 1.31–2.14; p = 0.009), ECOG-PS (OR: 20.01, 95% CI: 12.89–32.73; p < 0.0001), systemic therapy lines (OR: 1.22, 95% CI: 1.05–1.41; p = 0.012), and disease status (OR: 4.50, 95% CI: 3.12–6.48; p < 0.0001). These findings are presented in Supplementary Fig. S1. In the multivariable analysis, a high PROMISE score (OR: 3.32, 95% CI: 1.40–7.86; p = 0.006) and a high CTI score (OR: 2.85, 95% CI: 1.32–6.18; p = 0.008) were independent predictors of higher 90-day mortality, whereas a high GRIm score (OR: 1.83, 95% CI: 0.83–2.91; p = 0.07) was not significantly associated. A low PROMISE score (OR: 0.22, 95% CI: 0.10–0.49; p = 0.001) and a low CTI score (OR: 0.35, 95% CI: 0.17–0.73; p = 0.003) were independent predictors of lower 90-day mortality, whereas a low GRIm score (OR: 0.73, 95% CI: 0.47–1.20; p = 0.08) was not significantly associated. These findings are presented in Fig. 3.

The Spearman correlation analysis revealed significant associations among the PROMISE, CTI, and GRIm scores. The correlation coefficient between PROMISE and CTI scores was 0.60 (p < 0.0001). Similarly, the correlation between PROMISE and GRIm scores was 0.71 (p < 0.0001), and between CTI and GRIm scores, it was 0.58 (p < 0.0001).

**Fig. 4:**
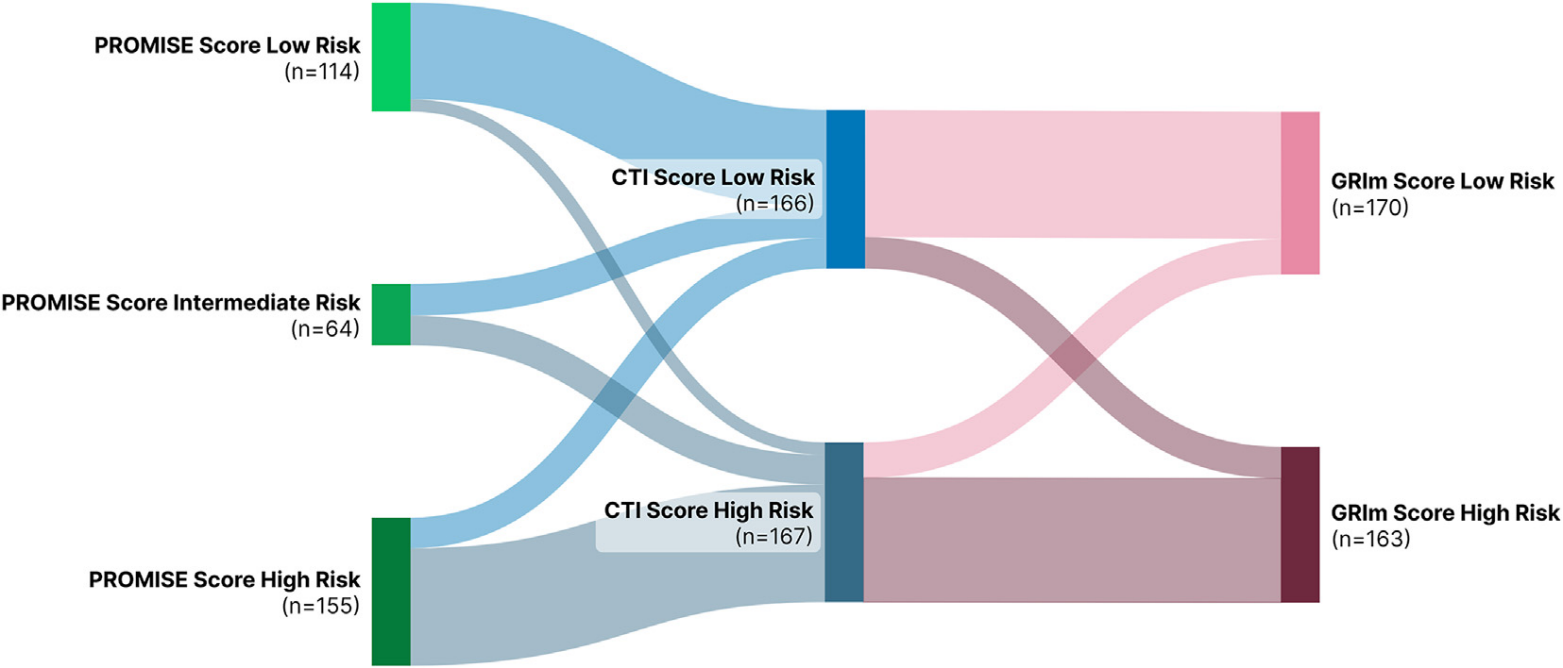
**Sankey diagram of risk classification by PROMISE, CTI, and GRIm scores (n = 333).** This figure illustrates transitions between risk strata across scoring systems.

Since CTI was calculated for all 333 patients, the GRIm and PROMISE scores were also assessed in the same patient cohort. Fig. 4 presents a Sankey diagram illustrating the transitions and consistency in risk classifications across these scoring systems, visualizing how patients were classified into different risk groups by each system.

**Fig. 5:**
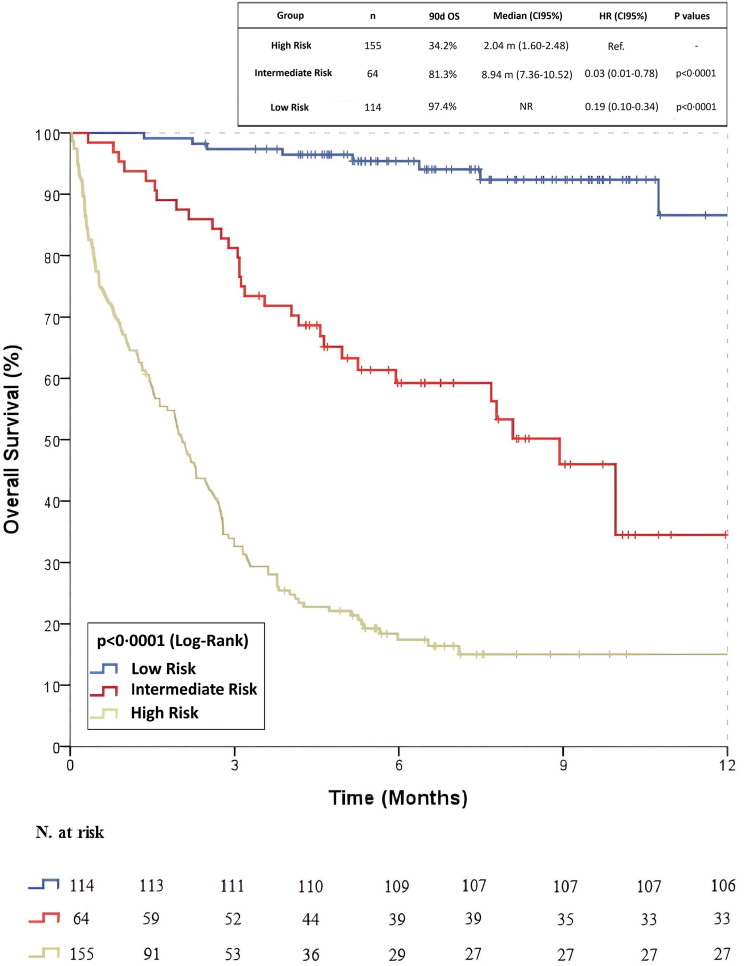
**Kaplan–Meier survival analysis based on PROMISE score in patients stratified by CTI score (n = 333).** Separate survival curves were plotted for CTI Score-low and CTI Score-high groups within each PROMISE risk category.

In this study, the 90-day mortality rate among 333 patients assessed using the CTI score was 35.1% (n = 117). When stratified using the PROMISE score, the low-risk group (n = 114) included 111 patients (97.4%) who survived and 3 patients (2.6%) who died within 90 days. In the intermediate-risk group (n = 64), 52 patients (81.3%) survived, while 12 patients (18.8%) died during the 90-day period. Among the high-risk group (n = 155), 53 patients (34.2%) survived, whereas mortality occurred in 102 patients (65.8%) within 90 days. Median OS for the overall cohort was 7.49 months (95% CI: 4.92–10.06). Kaplan–Meier survival curves are presented in Fig. 5.

### Predictive performance of the PROMISE-CTI **C**ombined score for 90-day mortality

**Fig. 6:**
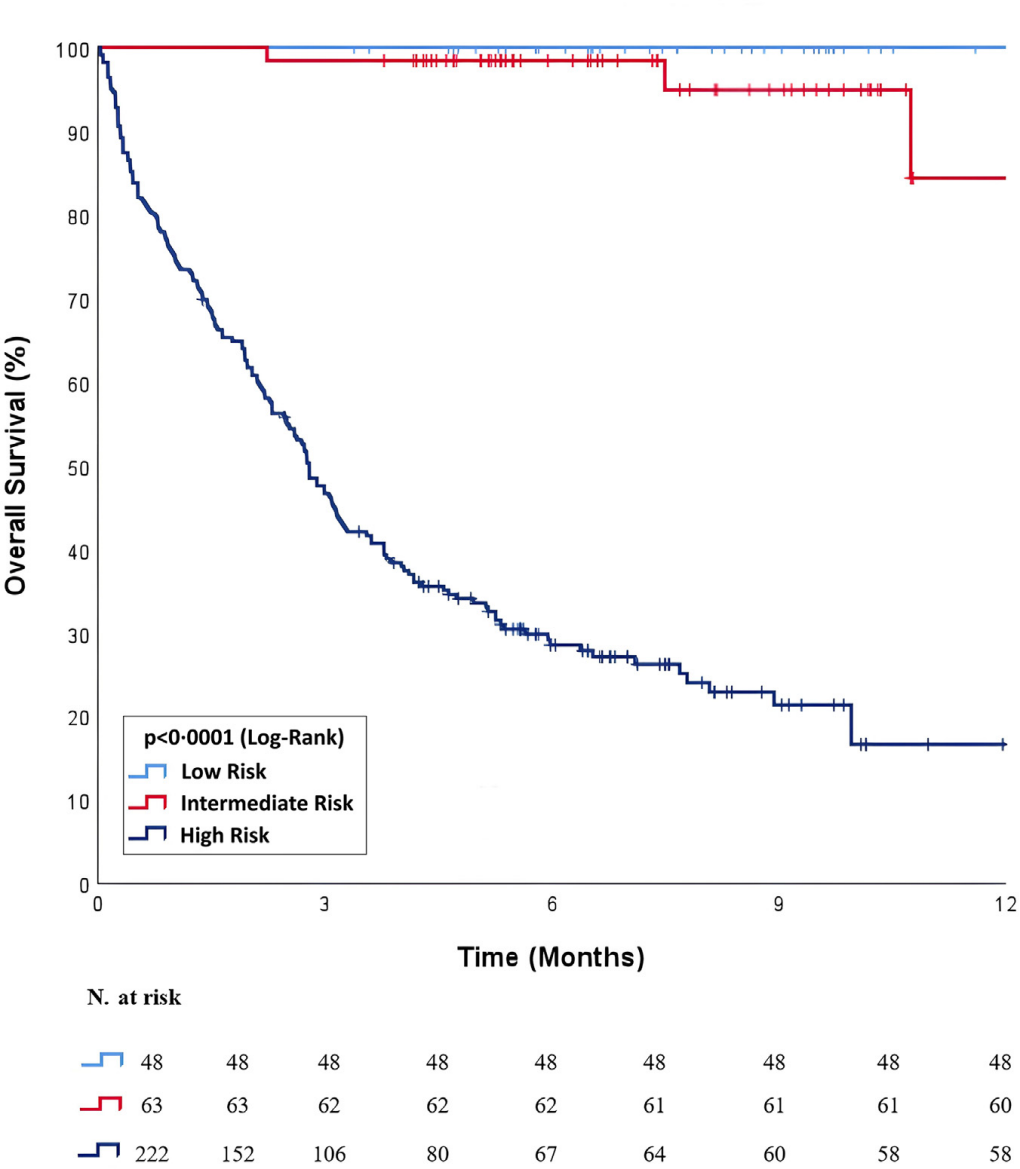
**Kaplan–Meier survival analysis stratified by the PROMISE–CTI Combined score (n = 333).** Survival curves were generated for low-, intermediate-, and high-risk groups as classified by the combined model.

In the 90-day mortality risk assessment of 333 patients using the PROMISE-CTI Combined score, 48 patients (14.4%) were classified as low risk, 63 patients (18.9%) as intermediate risk, and 222 patients (66.7%) as high risk. During the 90-day mortality follow-up, no deaths occurred in the low-risk group (0.0%), while 1 patient in the intermediate-risk group (1.6%) and 116 patients in the high-risk group (52.2%) died (p < 0.0001). Kaplan–Meier survival analysis findings are presented in Fig. 6.

**Fig. 7:**
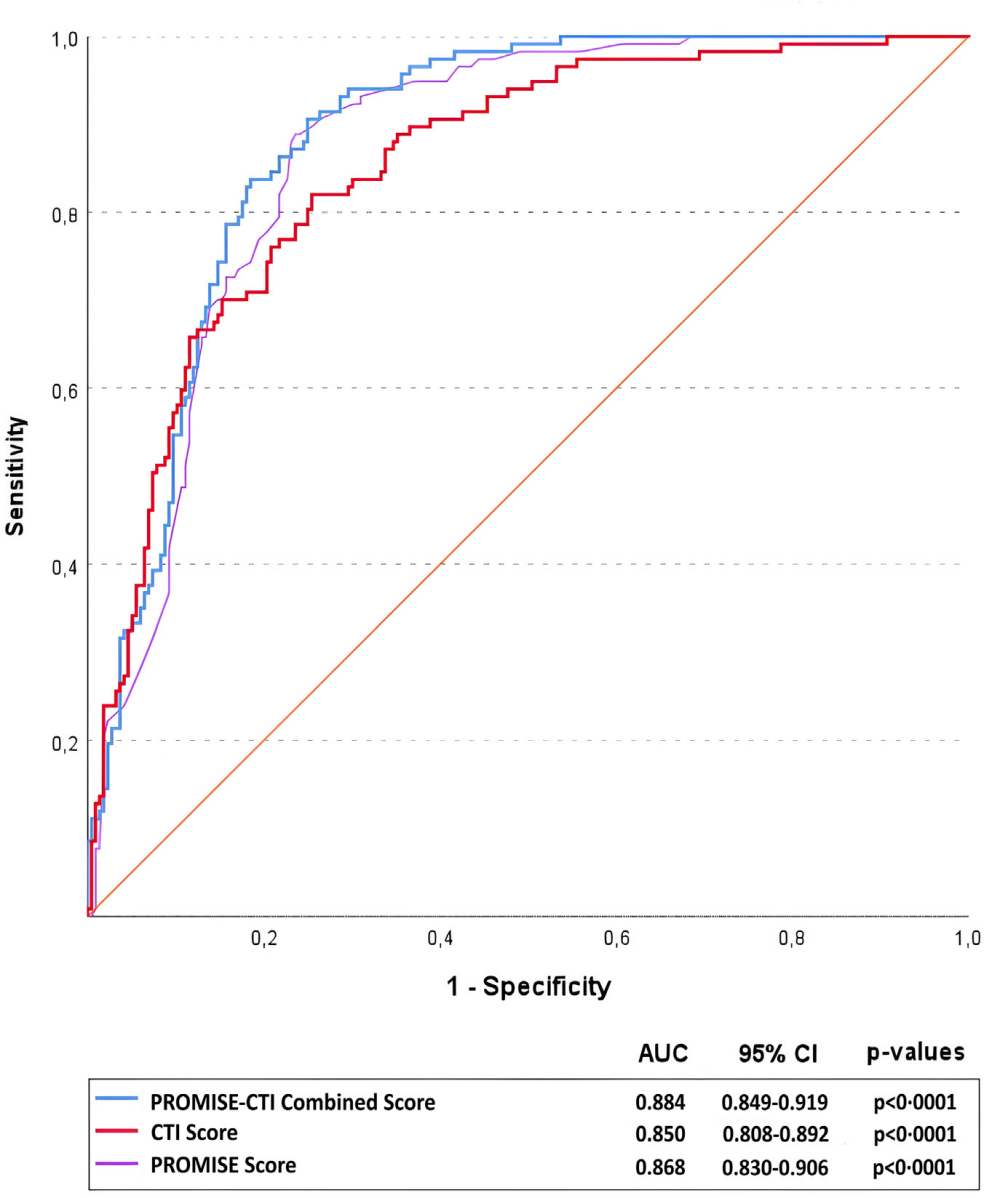
**Receiver operating characteristic (ROC) curves for PROMISE, CTI, and PROMISE–CTI Combined scores in predicting 90-day mortality.** Area under the curve (AUC) was calculated for each model.

The PROMISE-CTI Combined Score exhibited strong discriminative performance for predicting short-term mortality, with an AUC of 0.884 (95% CI: 0.849–0.919, p < 0.0001). The AUC values for the PROMISE and CTI scores were 0.868 (95% CI: 0.830–0.906, p < 0.0001) and 0.850 (95% CI: 0.808–0.892, p < 0.0001), respectively (Fig. 7). Previously validated cutoff values were applied for the PROMISE and CTI scores, while the optimal PROMISE-CTI threshold was derived from ROC analysis. The sensitivity, specificity, and predictive values of the PROMISE-CTI Combined Score are presented in Table 2. Model calibration analyses are presented in Supplementary Fig. S2. However, this strong performance should be interpreted with caution, as the PROMISE-CTI Combined Score was developed and validated using the same dataset, which may lead to optimistic estimates due to overfitting.

**Table 2:** Predictive performance of the PROMISE, CTI, and PROMISE-CTI Combined scores for 90-day mortality.^a^

Parameters (n = 333)	Sensitivity, (%)	Specificity, (%)	PPV, (%)	NPV, (%)	Accuracy, (%)
PROMISE Score	88.7	64.3	78.0	83.0	79.9
PROMISE Score (Low to Intermediate risk)	76.2	64.3	72.5	85.1	80.2
PROMISE Score (Intermediate to High risk)	82.5	69.4	75.3	87.3	82.7
CTI Score	88.2	67.5	79.1	81.4	81.0
PROMISE-CTI Combined score	92.4	81.1	85.3	89.6	86.7
PROMISE-CTI Combined score (Low to Intermediate risk)	80.3	71.5	77.8	84.0	81.2
PROMISE-CTI Combined score (Intermediate to High risk)	86.4	75.8	81.1	87.9	84.6

CTI score: CRP-Triglyceride-Glucose Index, n: sample size, NPV: Negative Predictive Value, PPV: Positive Predictive Value, PROMISE score: Prognostic Score for Hospitalized Cancer Patients.

a Risk classification was based on predefined cut-off values. For the PROMISE score, a low-to-intermediate transition occurred at 27.33%, and an intermediate-to-high transition at 53.04%. For the CTI score, a low-to-high transition occurred at 4.78. For the PROMISE-CTI Combined score, a low-to-intermediate transition occurred at 6.5, and an intermediate-to-high transition at 8.0.

**Fig. 8:**
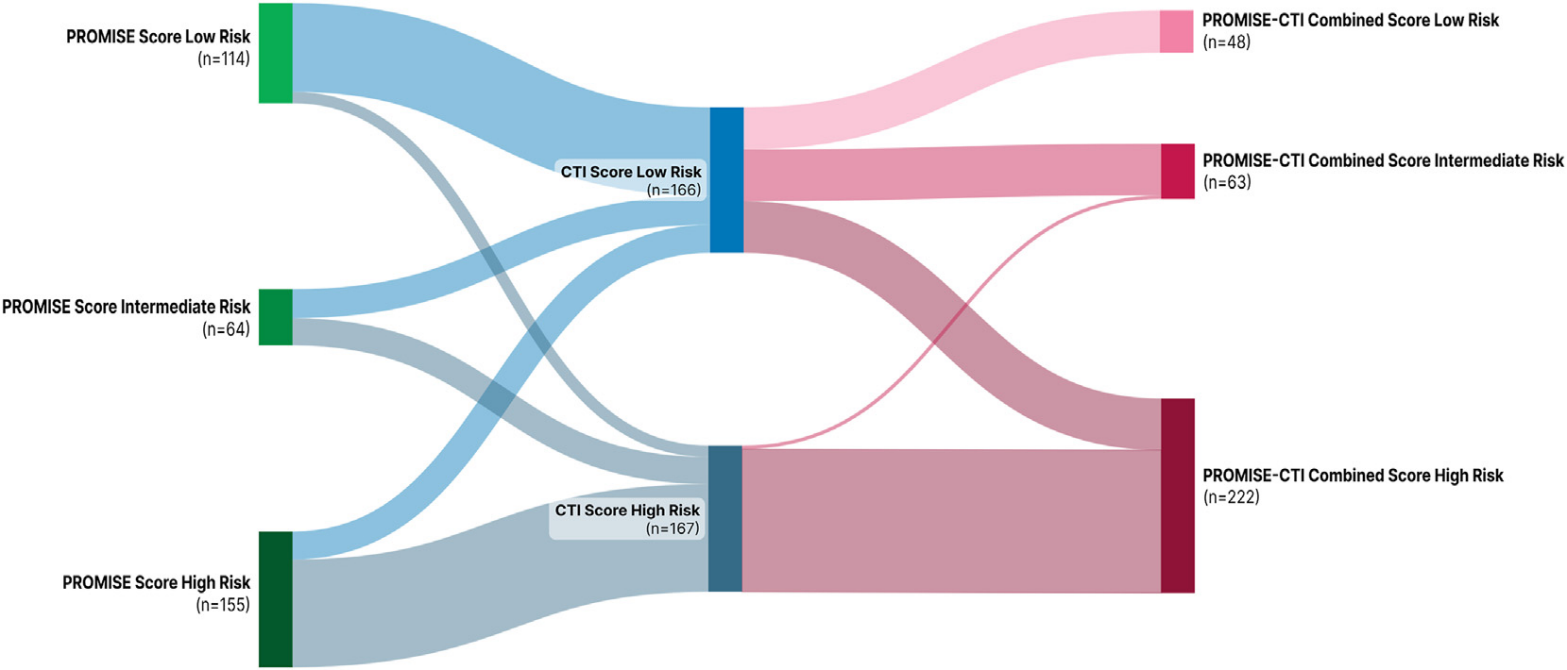
**Sankey diagram of PROMISE, CTI, and PROMISE–CTI Combined risk scores (n = 333).** This diagram visualizes concordance and discordance among the scoring systems.

Among the 333 patients, 108 (32.4%) were consistently classified as high risk across all three scoring systems (PROMISE, CTI, and PROMISE-CTI Combined Scores), while 93 (27.9%) remained persistently categorized as low risk. Fig. 8 illustrates a Sankey diagram depicting the transitions and concordance in risk classification across these scoring systems.

**Fig. 9:**
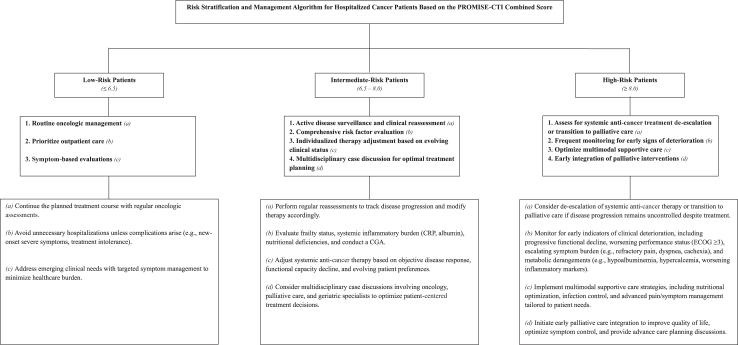
**Risk-stratified management algorithm for hospitalised cancer patients based on the PROMISE–CTI Combined score.** The algorithm outlines proposed clinical actions for each risk group.

The risk stratification and management algorithm based on the PROMISE-CTI Combined Score (Fig. 9) provides a structured framework for guiding oncologists in treatment planning, risk-adapted follow-up, and supportive care strategies for hospitalised cancer patients. Future multicentre prospective randomised trials are warranted to validate its clinical impact on survival outcomes, quality of life, and healthcare resource optimization.

### Subgroup analysis by cancer type

For lung cancer patients (n = 283), mortality rates based on the PROMISE score were 2.4% (n = 1) in the low-risk group, 3.6% (n = 2) in the intermediate-risk group, and 51.9% (n = 96) in the high-risk group (p < 0.0001). According to the PROMISE score, median OS was 2.83 months (95% CI: 2.37–3.29) in the high-risk group, while it was not reached in the low- and intermediate-risk groups. The GRIm score showed a 7.9% (n = 9) mortality rate in the low-risk group and 53.3% (n = 90) in the high-risk group (p < 0.0001). According to the GRIm score, median OS was 2.79 months (95% CI: 2.40–3.19) in the high-risk group and 12.25 months in the low-risk group (95% CI not estimable). Similarly, CTI score analysis (n = 62) revealed mortality rates of 9.4% (n = 3) in the low-risk group and 60.0% (n = 18) in the high-risk group (p < 0.0001). Based on the CTI score, median OS was 2.29 months (95% CI: 0.76–3.84) in the high-risk group, while it was not reached in the low-risk group (Supplementary Fig. S3).

For gastrointestinal cancer patients (n = 350), the PROMISE score demonstrated mortality rates of 6.0% (n = 5) in the low-risk group, 23.7% (n = 18) in the intermediate-risk group, and 63.2% (n = 120) in the high-risk group (p < 0.0001). According to the PROMISE score, median OS was 2.14 months (95% CI: 1.74–2.53) in the high-risk group and 5.45 months (95% CI: 4.18–6.73) in the intermediate-risk group, while it was not reached in the low-risk group. The GRIm scores indicated a 13.6% (n = 20) mortality rate in the low-risk group compared to 60.6% (n = 123) in the high-risk group (p < 0.0001). According to the GRIm score, median OS was 2.20 months (95% CI: 1.72–2.68) in the high-risk group and 9.30 months (95% CI: 8.12–10.48) in the low-risk group. The CTI scores (n = 95) showed mortality rates of 17.8% (n = 8) in the low-risk group versus 64.0% (n = 32) in the high-risk group (p < 0.0001). According to the CTI score, median OS was 1.94 months (95% CI: 1.09–2.79) in the high-risk group and 10.74 months (95% CI: 9.61–11.88) in the low-risk group (Supplementary Fig. S4).

For breast cancer patients (n = 92), the PROMISE score revealed 90-day mortality rates of 2.8% (n = 1) in the low-risk group, 21.4% (n = 3) in the intermediate-risk group, and 59.5% (n = 25) in the high-risk group (p < 0.0001). According to the PROMISE score, median OS was 2.04 months (95% CI: 1.17–2.90) in the high-risk group, while it was not reached in the low- and intermediate-risk groups. The GRIm score demonstrated mortality rates of 6.8% (n = 3) in the low-risk group compared to 54.2% (n = 26) in the high-risk group (p < 0.0001). According to the GRIm score, median OS was 2.23 months (95% CI: 1.08–3.39) in the high-risk group, while it was not reached in the low-risk group. CTI score analysis (n = 54) indicated mortality rates of 15.4% (n = 4) in the low-risk group versus 57.1% (n = 16) in the high-risk group (p < 0.0001). According to the CTI score, median OS was 2.04 months (95% CI: 0.84–3.23) in the high-risk group, while it was not reached in the low-risk group (Supplementary Fig. S5).

For patients with other cancers (n = 384), PROMISE score analysis showed no 90-day mortality in the low-risk group (n = 0), a mortality rate of 23.2% (n = 16) in the intermediate-risk group, and 60.8% (n = 121) in the high-risk group (p < 0.0001). According to the PROMISE score, median OS was 2.37 months (95% CI: 1.95–2.78) in the high-risk group and 5.95 months (95% CI: 4.64–7.25) in the intermediate-risk group, while it was not reached in the low-risk group. The GRIm scores revealed a 14.6% (n = 30) mortality rate in the low-risk group and 59.8% (n = 107) in the high-risk group (p < 0.0001). According to the GRIm score, median OS was 2.46 months (95% CI: 1.99–2.94) in the high-risk group, while it was not reached in the low-risk group. CTI score analysis (n = 122) demonstrated mortality rates of 6.3% (n = 4) in the low-risk group and 54.2% (n = 32) in the high-risk group (p < 0.0001). According to the CTI score, median OS was 2.76 months (95% CI: 2.11–3.41) in the high-risk group, while it was not reached in the low-risk group (Supplementary Fig. S6).

## Discussion

Unplanned hospitalisations in cancer patients constitute a significant component of oncology care. However, the predictive effectiveness of existing scoring systems in determining the prognosis of these patients remains suboptimal. This study evaluated the prognostic utility of the PROMISE, GRIm, and CTI scores in cancer patients experiencing unplanned hospitalisations. The findings indicated that both the PROMISE and CTI scores effectively stratify patients into high- and low-risk categories. Furthermore, the combination of the PROMISE and CTI scores in the PROMISE-CTI Combined score improved the identification of both 90-day mortality risk and the classification of patients into low- and high-risk groups more accurately. Subgroup analyses demonstrated that the PROMISE and CTI scores successfully identified 90-day mortality and low-risk patient groups within the subgroups of lung, gastrointestinal, breast, and other cancer types. The PROMISE score has been identified as the most accurate tool for predicting 90-day mortality compared to the GRIm and CTI scores. The PROMISE score has been shown to be suitable for integration into routine oncology practice due to its use of easily accessible clinical and laboratory data. Furthermore, its accuracy in identifying low-risk patients with a high negative predictive value underscores its potential as a valuable tool for treatment planning, palliative care, and critical decision-making processes in oncology. The integration of the CTI score into the PROMISE score has enhanced the predictive accuracy for 90-day mortality, demonstrating that this combined approach could serve as a valuable metric for assessing patients in terms of treatment, supportive care, and clinical decision-making.

In cancer patients, particularly those requiring hospitalisation at advanced stages, 90-day mortality is regarded as a critical prognostic metric that informs clinical decision-making and directly influences patient outcomes.^25^ However, the heterogeneity of patient populations and methodological differences across studies (e.g., tumour types, treatment strategies, nutritional status, and methods for assessing inflammatory markers) pose significant challenges to deriving generalized conclusions.^26^ Most studies in the literature have primarily investigated 90-day mortality in the context of postoperative periods or intensive care unit admissions.^11^^,^^25^^,^^27^^,^^28^ To address this gap, our study focused on analysing unplanned hospitalisations in advanced-stage cancer patients. Various prognostic factors associated with 90-day mortality have been identified in the literature. Studies conducted in the postoperative period have reported strong associations between 90-day mortality and advanced age, poor ECOG-PS (≥2), the presence of sarcopenia, disease progression status, and frailty, particularly among geriatric patients.^29^, ^30^, ^31^, ^32^, ^33^ Additionally, studies on advanced-stage cancer patients presenting to emergency departments have reported significant associations between low albumin levels, low BMI, and 90-day mortality.^34^ In our study, factors included in the PROMISE score, such as poor ECOG-PS (≥2), disease progression status, and low albumin levels, were found to be significantly associated with 90-day mortality. These findings align with previous studies and further support the predictive validity of the PROMISE score. However, advanced age and low BMI were not significantly associated with 90-day mortality in our study, which may be attributed to patient heterogeneity and differences in population characteristics. Due to the retrospective design of our study and data limitations, sarcopenia could not be evaluated. Similarly, frailty analysis was not conducted as our patient population was not restricted to geriatric individuals. Our findings demonstrate that the PROMISE score is an effective tool for predicting 90-day mortality and supports its utility as a readily accessible prognostic marker.

The PROMISE score has been shown to be effective in predicting 90-day mortality in cancer patients with unplanned hospital admission. In the study by Mirallas et al., survival rates for low, intermediate, and high-risk groups were reported as 83.6%, 68.0%, and 33.4%, respectively. Independent prognostic factors identified included an ECOG-PS of ≥2, progressive disease status, elevated LDH, and low albumin levels.^14^ Our findings support the effectiveness of the PROMISE score in distinguishing between low- and high-risk groups. Furthermore, like the results reported by Mirallas et al., our subgroup analyses revealed that the PROMISE score effectively stratifies low- and high-risk patient groups across various cancer types, including lung, breast, gastrointestinal, and others. However, the differences in survival rates across risk groups may be attributed to variations in patient demographics, disparities in palliative care infrastructure across countries, and unequal access to healthcare services. Furthermore, our study evaluated a nutritional marker, the CTI score, alongside the PROMISE score, and demonstrated that the combination of these two metrics significantly improved prognostic accuracy compared to either score used alone. This novel integrative approach not only enhances the prediction of 90-day mortality but also provides a more comprehensive evaluation of patient outcomes, representing a distinctive and valuable contribution to the existing body of literature. Additionally, our study demonstrates that the PROMISE score is successfully applicable in two distinct populations and is effective across a broad patient cohort. The PROMISE score has the potential to become a standard tool in multidisciplinary oncology practice, facilitating optimized treatment decisions and palliative care processes. If validated through multicentre prospective studies, the PROMISE score may establish itself as a new standard for evaluating 90-day mortality risk in oncology care.

The GRIm score has been described by Bigot et al., as a meaningful tool reflecting inflammatory processes.^35^ Its prognostic significance has been particularly emphasized in patients with advanced malignancies or severe cancer-related cachexia, where systemic inflammation plays a prominent role. The GRIm score has been highlighted as an important tool for identifying patients with poor prognoses in specific groups.^21^^,^^22^ However, unlike these studies, our study is the first to evaluate 90-day mortality prediction in hospitalised cancer patients from this perspective. The findings of our study revealed that the GRIm score did not emerge as a strong predictor of 90-day mortality. This can be attributed to the superiority of more comprehensive and robust prognostic tools, such as the PROMISE and CTI scores. Nevertheless, the GRIm score may still hold value as a prognostic tool, particularly in future research focusing on specific patient populations. The CTI score has emerged as a strong prognostic tool that reflects both nutritional and inflammatory processes. Ruan et al. demonstrated that the CTI score effectively predicts 90- and 180-day mortality by capturing nutritional and inflammatory statuses in patients with cancer cachexia.^23^^,^^24^ Furthermore, a meta-analysis by Rinninella et al., systematically emphasized the prognostic significance of nutritional interventions in improving outcomes for cancer patients.^36^ Similarly, Mayengbam et al., highlighted the role of metabolic factors, particularly cholesterol, in tumour progression.^37^ This finding aligns with the CTI score’s framework, which combines nutritional and inflammatory parameters to enhance its prognostic utility. The impact of nutritional status on short-term mortality has been demonstrated in numerous studies and our study found the CTI score to be a strong predictor of mortality, even in a more limited patient population. Albumin, a key component of the PROMISE score, has been recognized as a nutritional marker. However, as a negative acute-phase reactant, albumin is known to be influenced by various systemic factors, which may limit its prognostic utility.^38^ Based on our findings, we propose that integrating an objective nutritional parameter such as the CTI score into the PROMISE framework has the potential to establish a new standard for evaluating low-risk patients. Future large-scale prospective studies are needed to comprehensively validate the prognostic value of PROMISE and CTI scores in predicting cancer mortality.

This study has certain limitations. The retrospective design restricts the ability to assess temporal changes and establish causality, while the single-centre nature of our study may impact the generalizability of the findings. However, Ankara Etlik City Hospital, as a major cancer reference centre, provides care for a highly diverse oncologic population, encompassing various cancer types, treatment backgrounds, and clinical conditions. While this heterogeneity enhances external validity, further validation in multicentre settings is warranted. Prospective, randomised, multicentre studies are essential to assess the broader applicability of these findings across different healthcare systems. In addition to internal validation, external validation across independent cohorts is necessary to confirm the robustness and generalizability of the PROMISE-CTI Combined Score. Given these limitations, careful consideration is required to ensure the optimal application of the PROMISE-CTI Combined score in real-world oncologic settings. A potential limitation of this study is the possibility of misclassification when applying the PROMISE-CTI Combined score. Although the score has demonstrated strong prognostic accuracy, certain subgroups may be affected by classification errors. Older adults with multiple comorbidities or low BMI might be underestimated in terms of risk, as some underlying prognostic determinants may not be fully captured within the model. Conversely, patients experiencing acute inflammatory responses or transient metabolic disturbances may be overclassified into the high-risk category, potentially leading to overtreatment. Additionally, the possibility of dividing the dataset into training and validation cohorts for internal validation was considered. However, given the limited number of patients eligible for CTI score calculation—primarily due to incomplete laboratory data—such an approach was deemed statistically suboptimal, as it would reduce power and increase the risk of overfitting.^39^ It should also be acknowledged that the PROMISE-CTI Combined Score was both developed and tested within the same dataset, which may result in optimistic estimates of predictive accuracy. This internal validation process introduces a natural advantage for the combined score. Beyond internal validation, external validation in independent multicentre cohorts is required to ensure the broader applicability and reliability of the PROMISE-CTI Combined Score across diverse oncologic populations. According to the results of our study, the 90-day mortality rate was higher compared to the study by Mirallas et al., in which the PROMISE score was developed.^14^ This difference may primarily be attributed to variations in access to palliative care services in our country. In our healthcare system, patients requiring palliative care are often admitted to oncology clinics rather than specialized palliative care centres. This factor should be considered when interpreting the results. Advanced machine learning (ML) and artificial intelligence (AI)-driven models have shown promise in oncology, particularly in non-linear risk prediction. However, the current study does not incorporate these methodologies, which may limit its adaptability in dynamic clinical settings. Future research should explore the integration of AI-based tools to refine risk stratification, reduce misclassification errors, and enhance real-time clinical decision support.^40^^,^^41^ These limitations may restrict the broader interpretation of our findings. However, this study also has significant strengths. It is among the first to systematically evaluate the prognostic value of CTI and GRIm scores in predicting 90-day mortality in advanced-stage hospitalised cancer patients. The integrated evaluation of nutritional and inflammatory parameters alongside the PROMISE score demonstrated success in predicting short-term mortality and provides a robust foundation for future research. Moreover, our analysis of different cancer types and their association with 90-day mortality underscores the broader applicability of these scores across various malignancies. The PROMISE score, validated in this study for the first time in hospitalised cancer patients, has demonstrated its utility as a powerful tool for identifying low-risk groups. This holds potential for improving treatment strategies, advancing standards in oncologic care and follow-up, and informing clinical decision-making processes. Finally, the large sample size of our study enhances the generalizability of our findings and provides a meaningful contribution to addressing existing gaps in the literature.

This study demonstrates the robust prognostic performance of the PROMISE score in predicting 90-day mortality among advanced-stage hospitalised cancer patients and its exceptional ability to accurately identify low-risk patient groups. Furthermore, the integration of the CTI score into the PROMISE score in the form of the PROMISE-CTI Combined score significantly improved the identification of both high-risk and low-risk patients increasing the accuracy of the tool by 7.8%, enhancing its prognostic performance in predicting 90-day mortality. These findings highlight the clinical utility of the PROMISE-CTI Combined score as a valuable tool for improving prognostic risk stratification and guiding personalized treatment strategies in oncology. By integrating the CTI score into the PROMISE score, the PROMISE-CTI Combined score incorporates both inflammatory and nutritional markers, allowing for more precise identification of both high- and low-risk patients compared to its individual components. Given the prognostic performance of the PROMISE-CTI Combined score, there is a clear need for prospective, multicentre validation studies to confirm its applicability across diverse oncologic populations. Furthermore, randomised, multicentre, and prospective studies are warranted to assess the generalizability of the model across different cancer types, treatment settings, and healthcare systems. To ensure robust validation, the establishment of an independent external validation cohort in a future prospective study is of critical importance. This would allow for the refinement of cutoff values and the evaluation of the model’s performance beyond the current dataset in real-world clinical scenarios. However, it should be noted that the PROMISE-CTI Combined Score was developed and validated within the same dataset, which may have led to an overestimation of its predictive performance. Therefore, external validation is crucial to assess its true clinical utility across independent populations. These findings suggest that the PROMISE-CTI Combined score may represent a novel prognostic approach for predicting 90-day mortality in hospitalised cancer patients, with the potential to enhance oncologic care by optimizing treatment pathways, enabling evidence-based risk assessment, and improving clinical decision-making.

## Contributors

GCU, OM, RD, and OS conceptualised the study and drafted the initial manuscript. GCU and OM performed the data analysis and interpretation. Data collection was conducted by GCU. KB, BMC, EY, JRB, SK, VNG, SEY, KSV, DGP, APG, CST, ÖBCÖ, SS, and RD contributed to literature review, clinical interpretation, and manuscript revisions. APG, CST, KSV, and VNG participated in statistical review and data visualisation. OS accessed and verified all underlying data and had final responsibility for the decision to submit for publication. All authors critically reviewed and approved the final version of the manuscript.

## Data sharing statement

The dataset analysed in this study is not publicly available to protect patient confidentiality. However, de-identified data may be shared upon reasonable request following approval from the Ankara Etlik City Hospital Ethics Committee and mutual agreement with the corresponding author. All data analyses were independently verified by GCU and OS, in accordance with The Lancet Regional Health—Europe’s data sharing policy.

## Declaration of interests

The authors declare no competing interests. No funding, article processing charges, or material support were received for the conduct of this study. All authors have completed the ICMJE disclosure form, and any potential conflicts have been transparently declared.
