# Supplementary material for: Prediction of 90-day mortality among cancer patients with unplanned hospitalisation: a retrospective validation study of three prognostic scores
Source: Lancet Reg Health Eur. 2025 May 8;54:101317. doi: 10.1016/j.lanepe.2025.101317 (PMC12266187; doi:10.1016/j.lanepe.2025.101317)
Supplement: Supplementary Figures S1–S6 and Table S1 [file mmc1.docx]

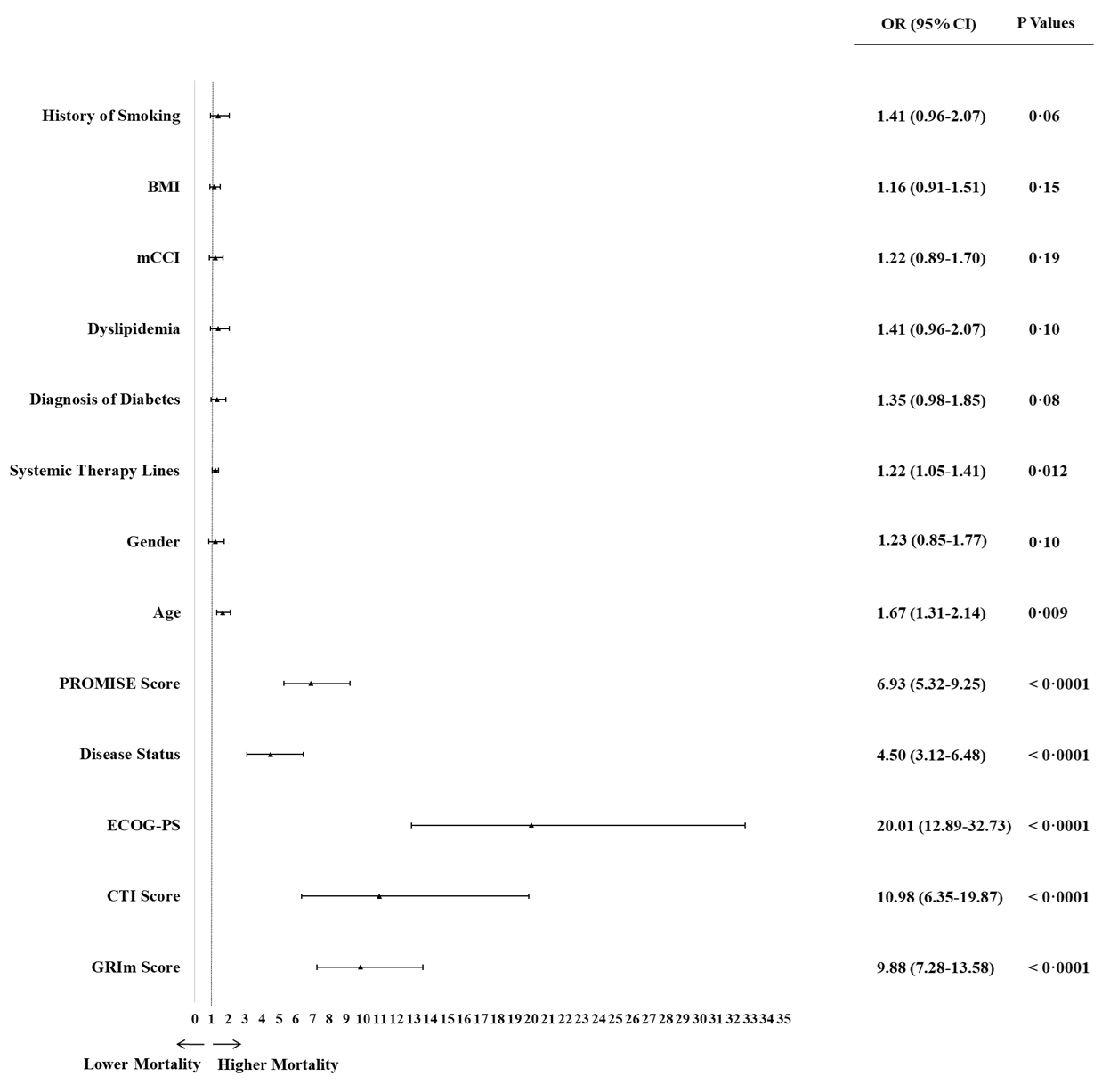


**Figure S1. Univariable Analysis of Factors Associated with 90-Day Mortality**

**Abbreviations: BMI:** Body Mass Index, **CI:** Confidence Interval, **CTI Score:** CRP-Triglyceride-Glucose Index, **ECOG-PS:** Eastern Cooperative Oncology Group Performance Status, **GRIm Score:** Gustave Roussy Immune Score,, **mCCI:** Modified Charlson Comorbidity Index, **OR:** Odds Ratio, **PROMISE Score:** Prognostic Score for Hospitalized Cancer Patients.


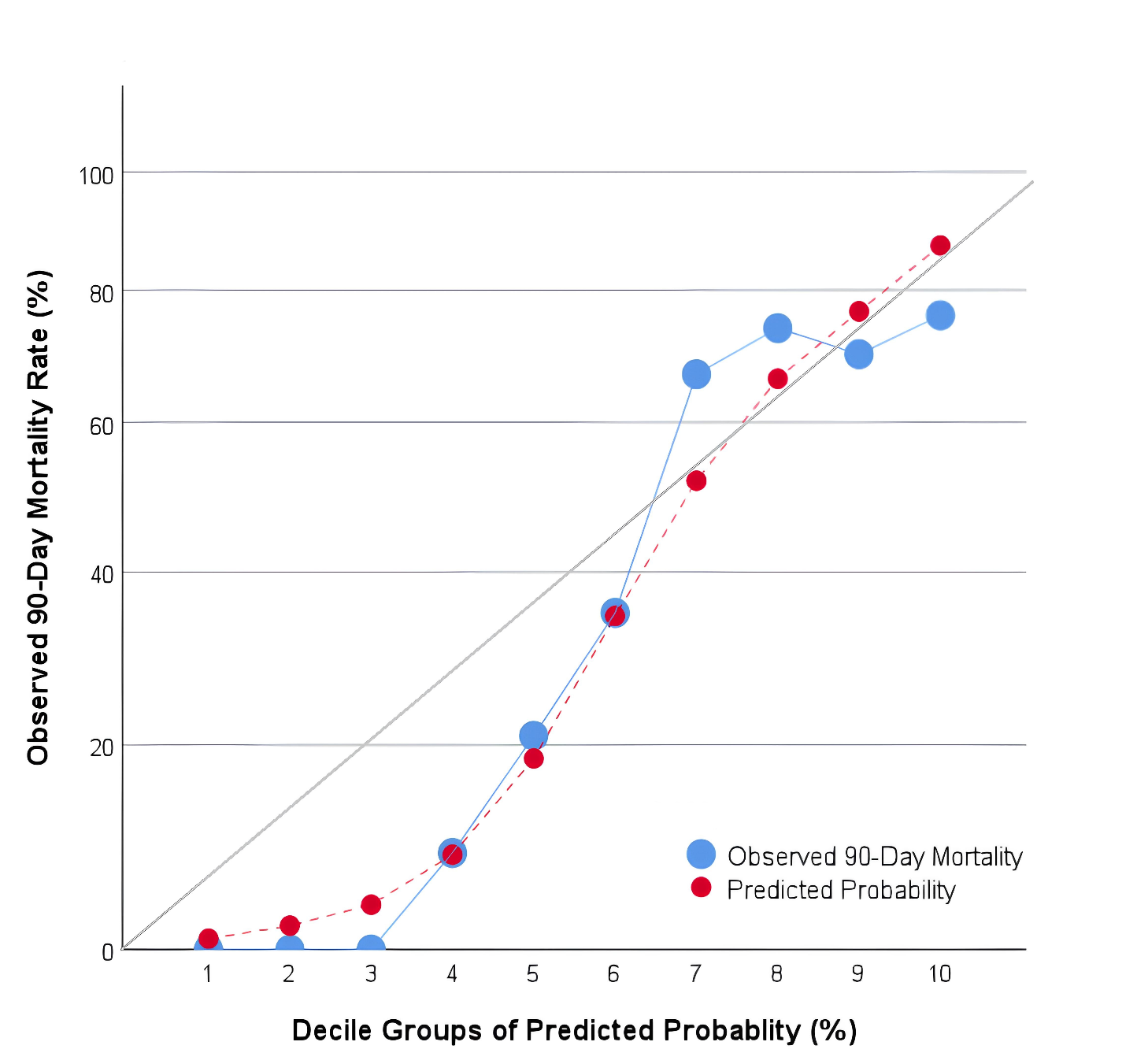


**Figure S2. Calibration Plot of the PROMISE-CTI Combined Score for 90-day Mortality Prediction**

Logit-transformed predicted probabilities were analyzed using linear regression with the PROMISE-CTI Combined Score as the independent variable (adjusted R² = 0.854, B = 1.571, SE = 0.036, p<0·0001). Model fit was assessed using ANOVA (F = 1937.915, p<0·0001) and the intercept was estimated at-4.713 (SE = 0.06). Calibration performance was evaluated using the Hosmer-Lemeshow test (p = 0·24), Nagelkerke R² (0.541), and Brier Score (0.1317).

**Abbreviations: CTI Score:** CRP-Triglyceride-Glucose Index, **PROMISE Score:** Prognostic Score for Hospitalized Cancer Patients


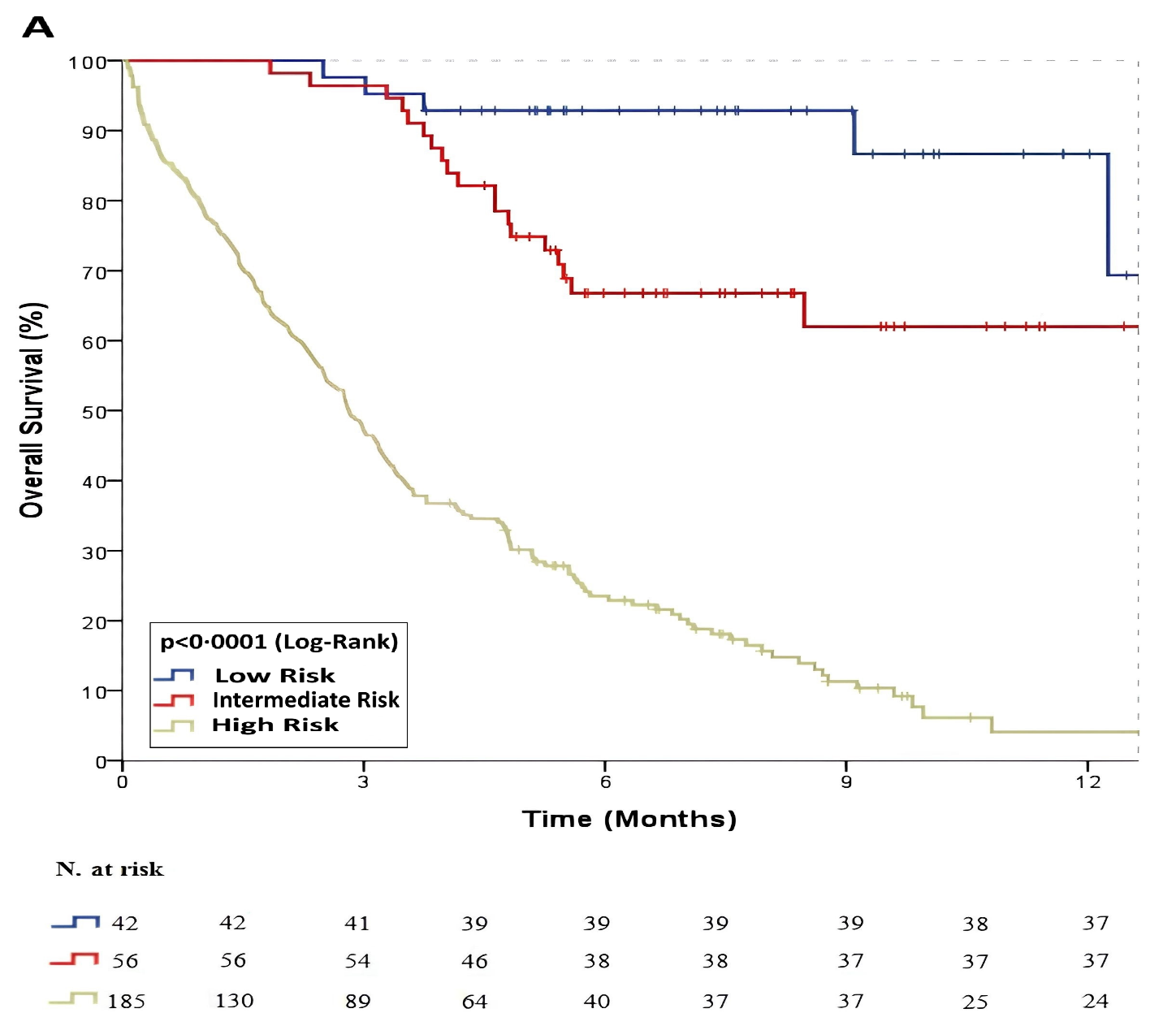


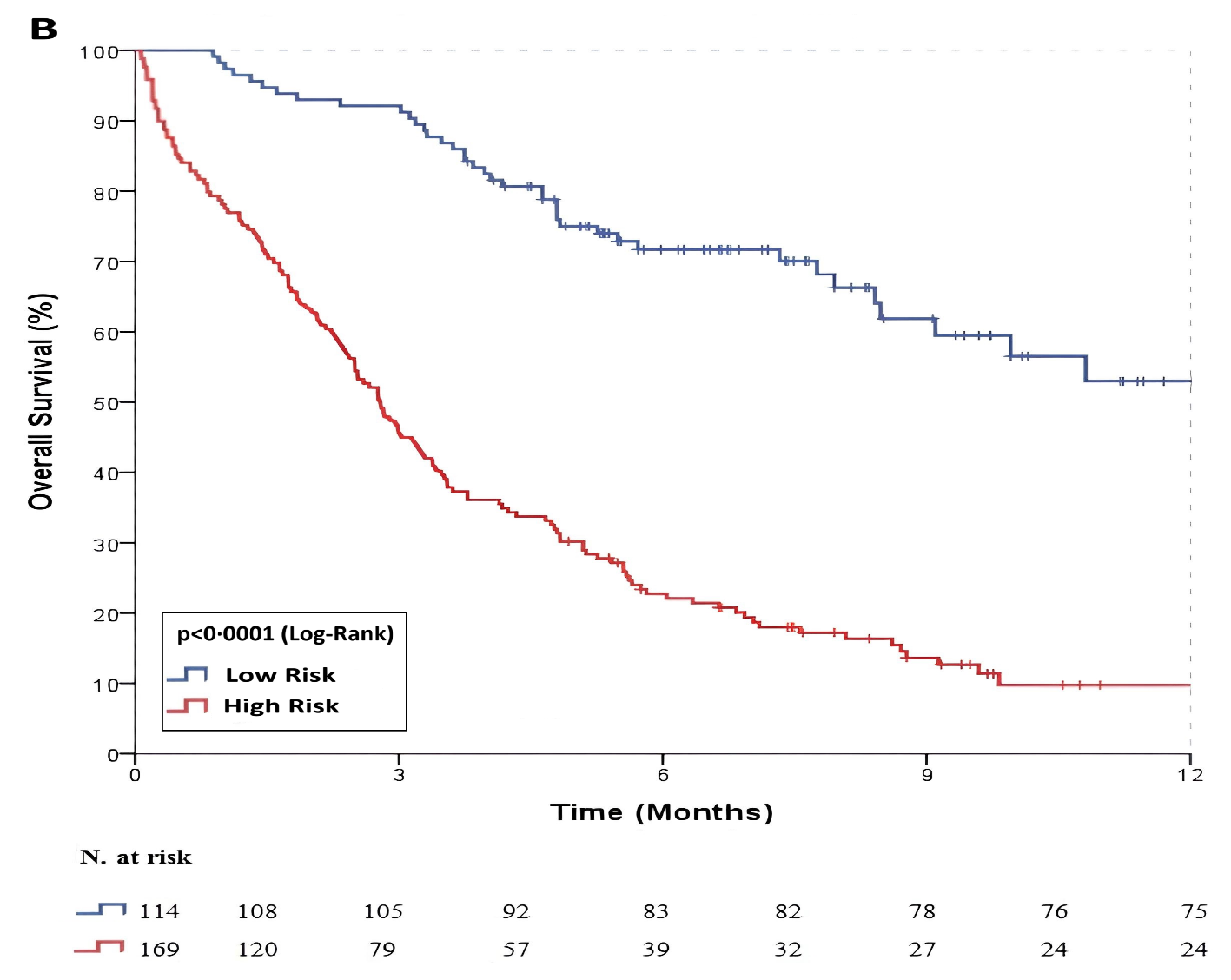


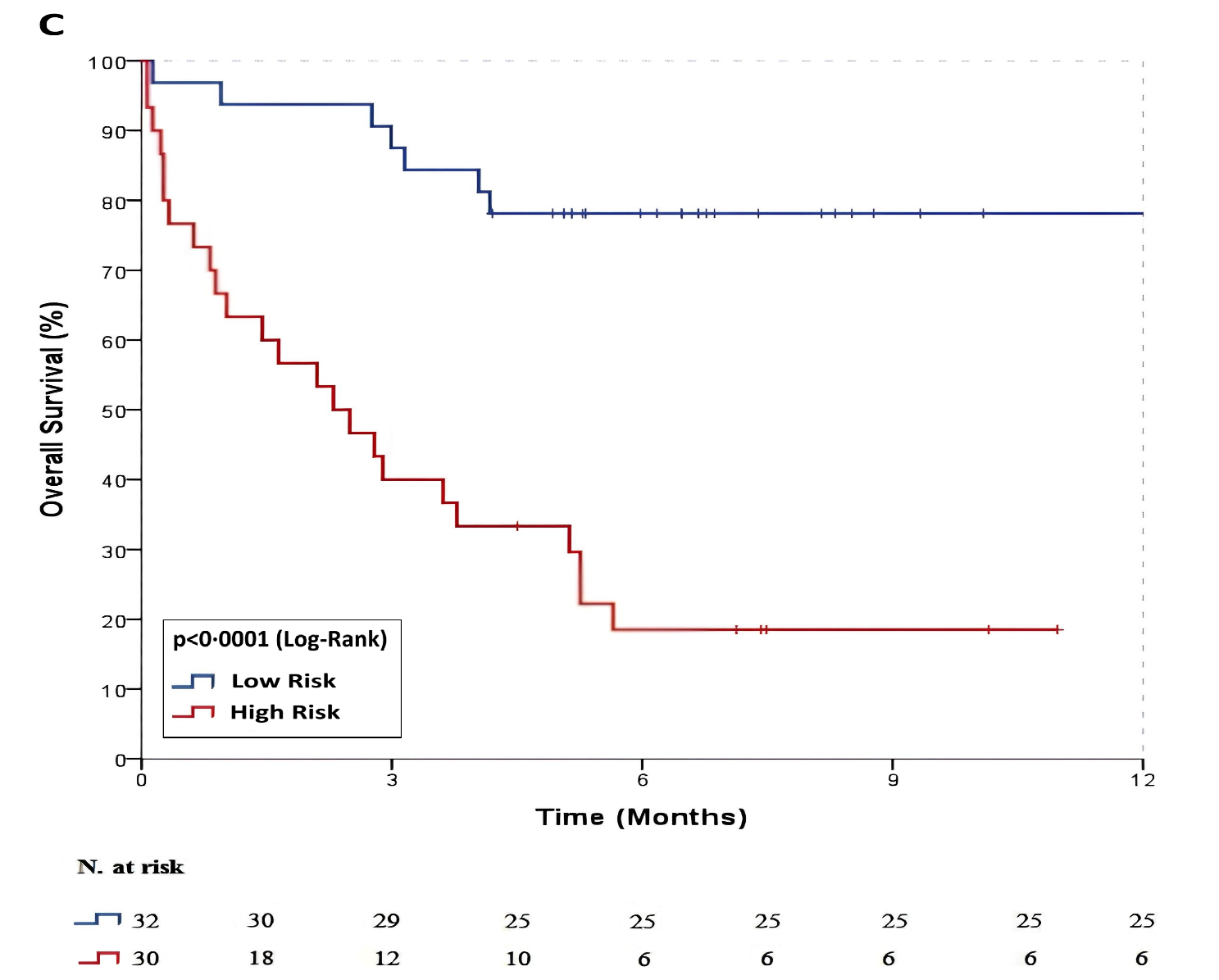


**Figure S3. Kaplan-Meier Survival Analysis in Lung Cancer Patients Stratified by Risk Scores**

(A) PROMISE score-based survival stratification (n=283) (B) GRIm score-based survival stratification (n=283) (C) CTI score-based survival stratification (n=62). The log-rank test was used to compare survival differences between risk groups.

**Abbreviations: CTI Score**: CRP-Triglyceride-Glucose Index, **GRIm Score**: Gustave Roussy Immune Score, **n:** sample size, **PROMISE Score**: Prognostic Score for Hospitalized Cancer Patients.


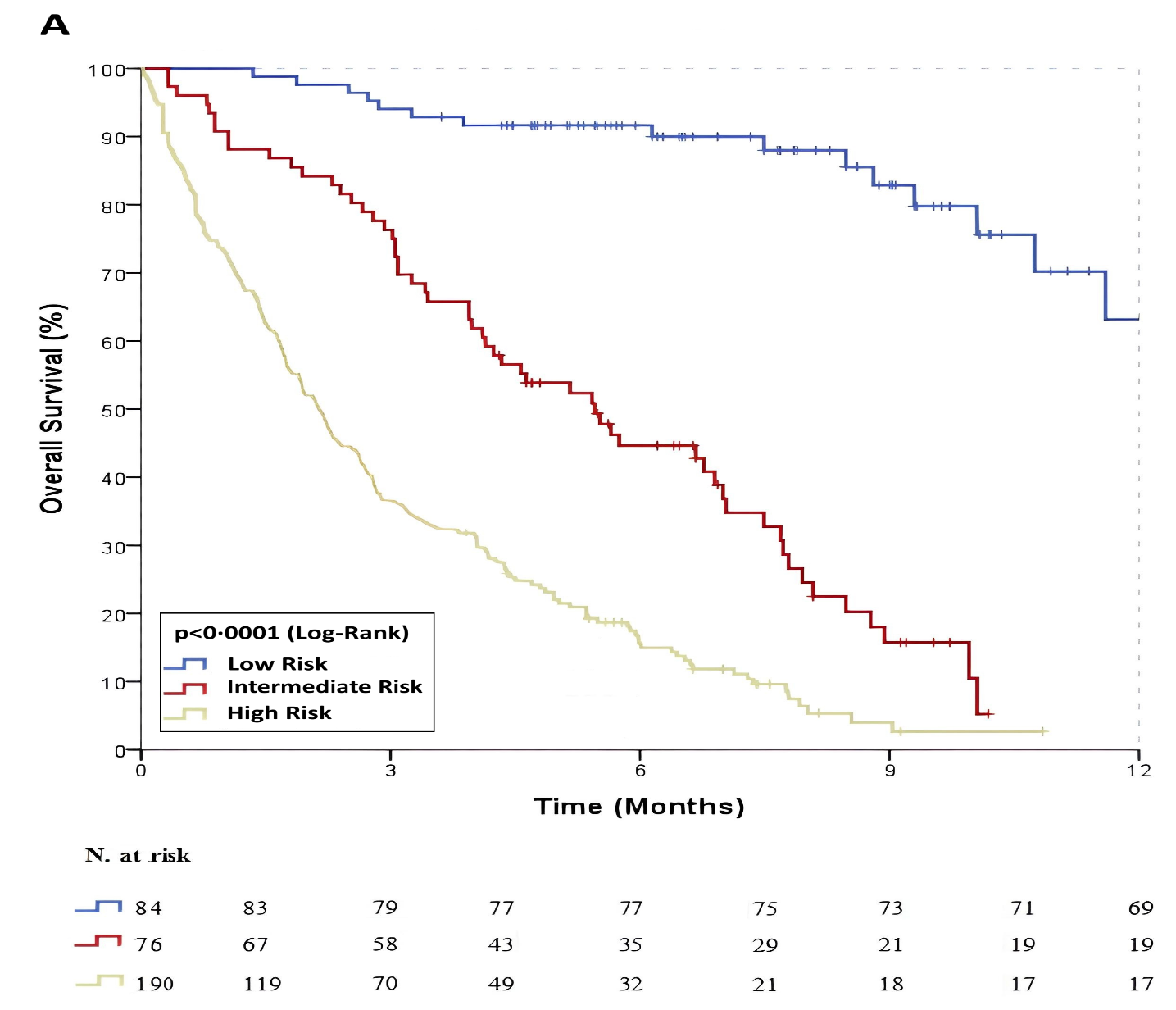


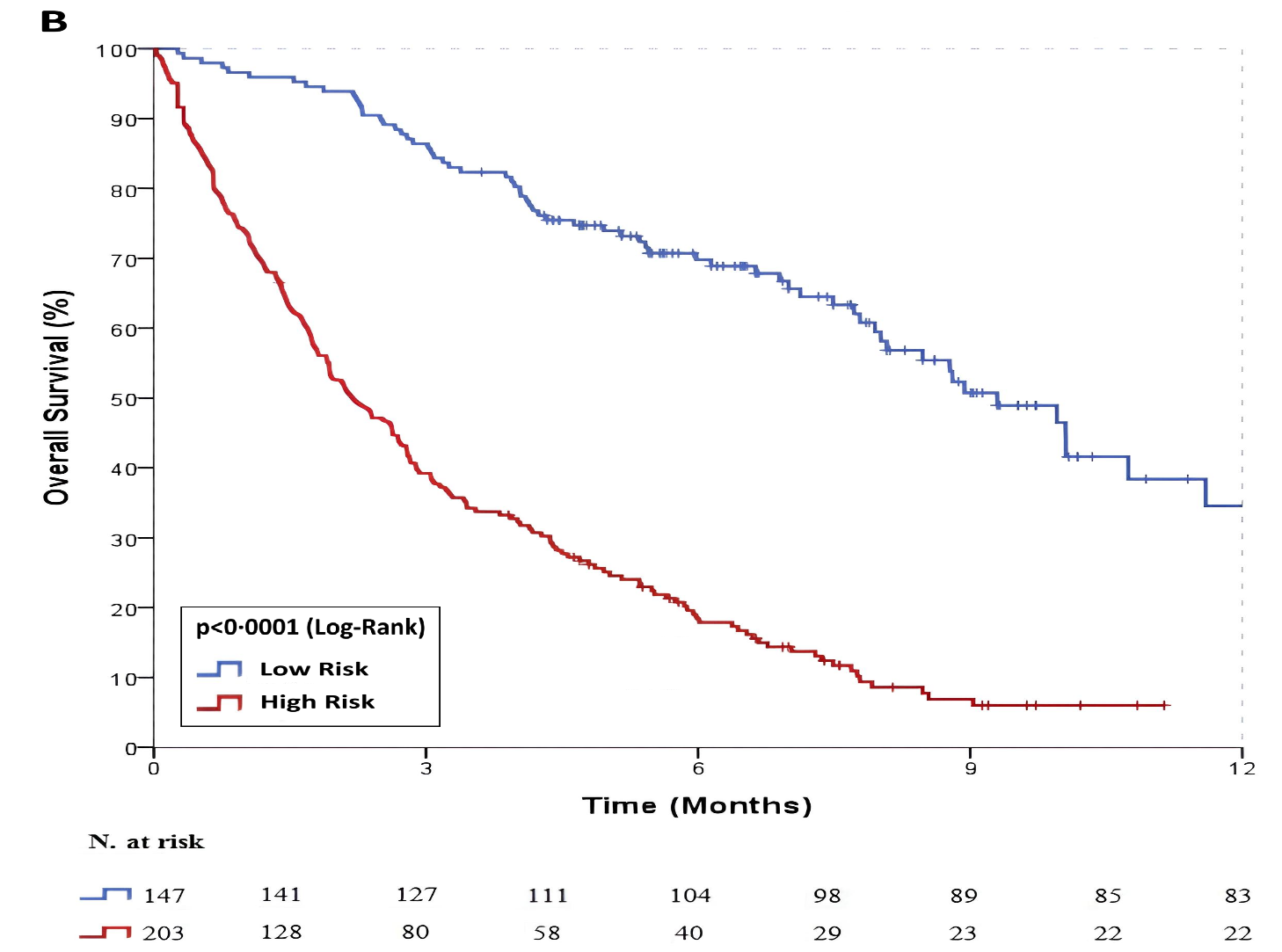


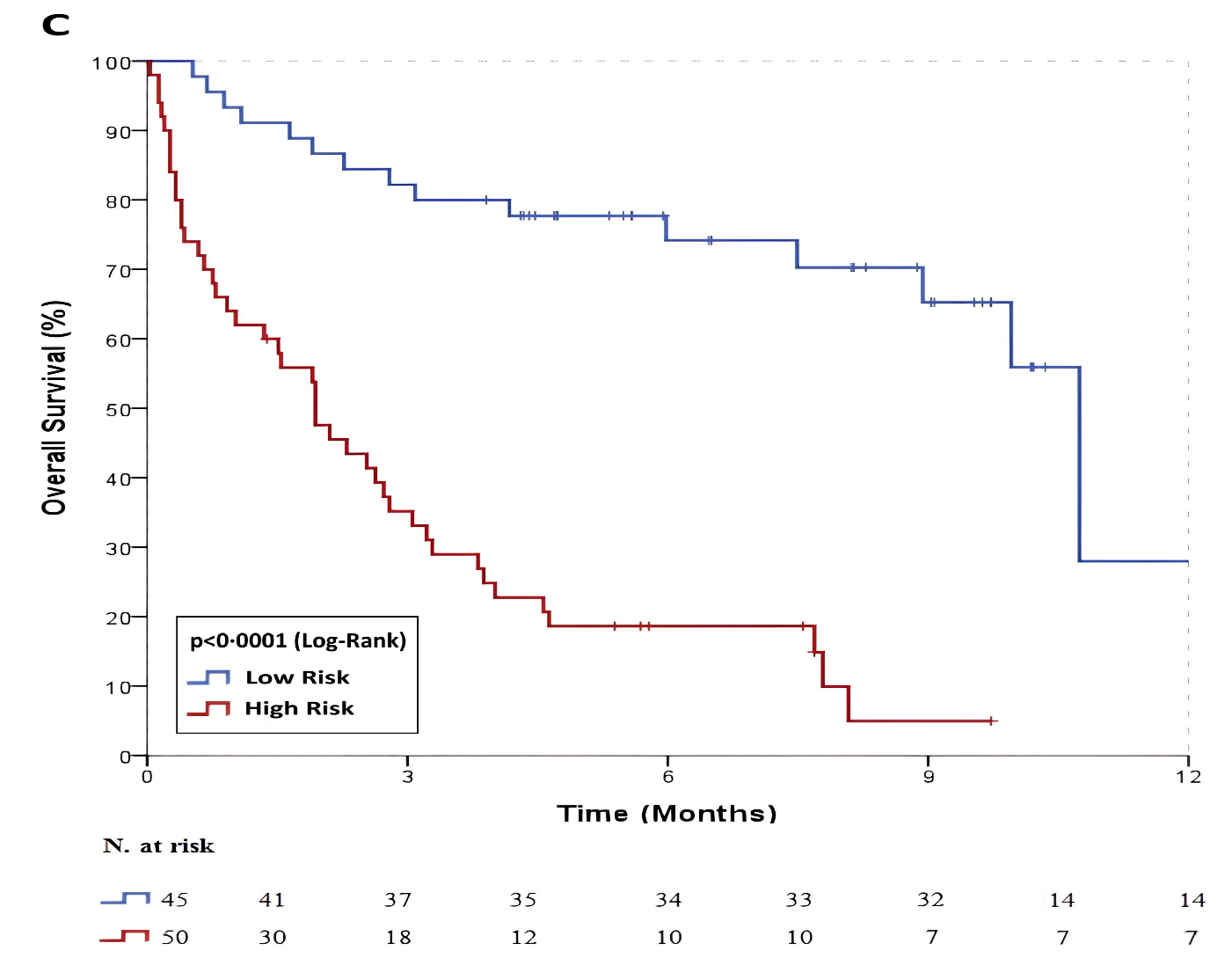


**Figure S4. Kaplan-Meier Survival Analysis in Gastrointestinal Cancer Patients Stratified by Risk Scores**

(A) PROMISE score-based survival stratification (n=350) (B) GRIm score-based survival stratification (n=350) (C) CTI score-based survival stratification (n=95). The log-rank test was used to compare survival differences between risk groups.

**Abbreviations: CTI Score**: CRP-Triglyceride-Glucose Index, **GRIm Score**: Gustave Roussy Immune Score, **n:** sample size, **PROMISE Score**: Prognostic Score for Hospitalized Cancer Patients.


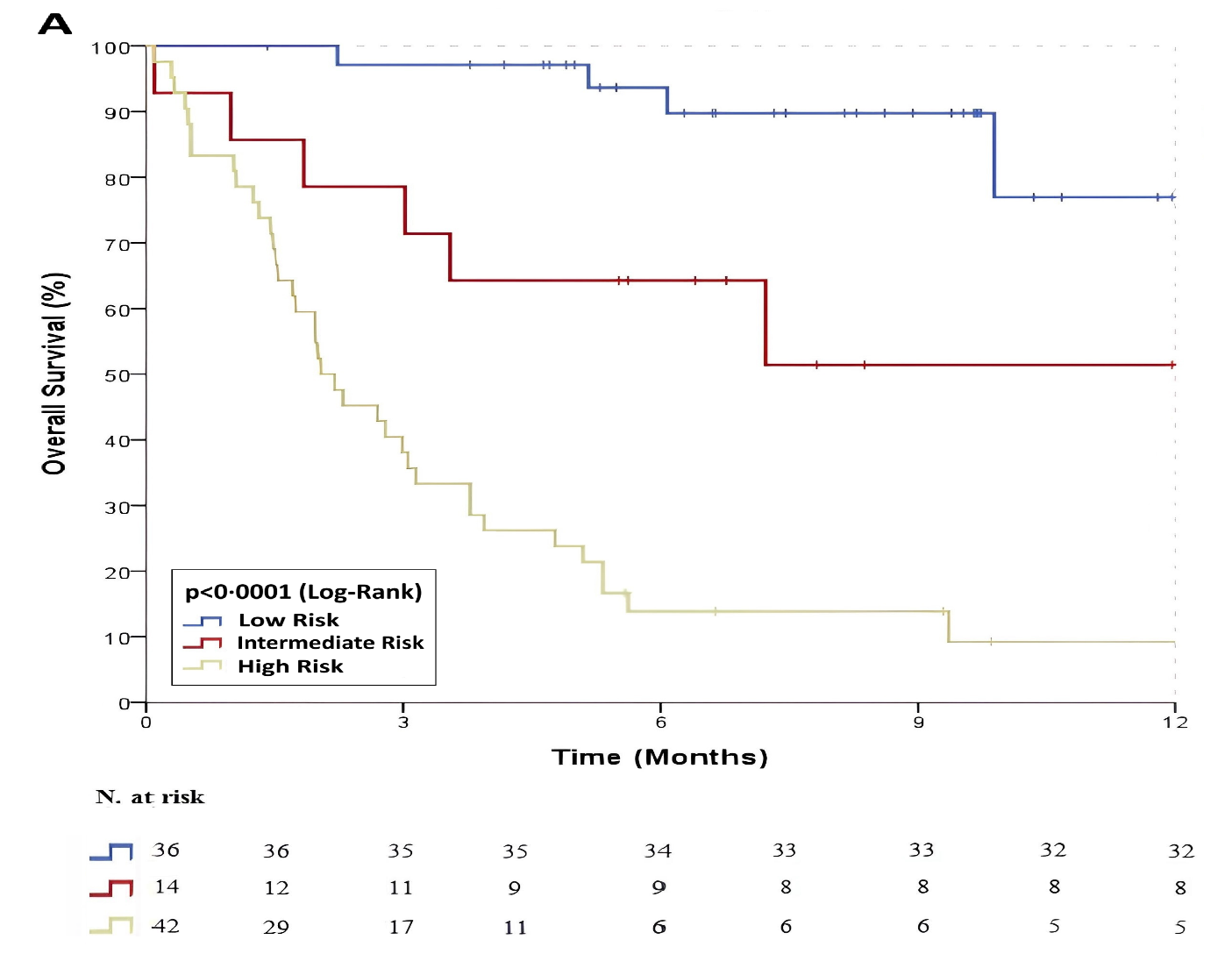


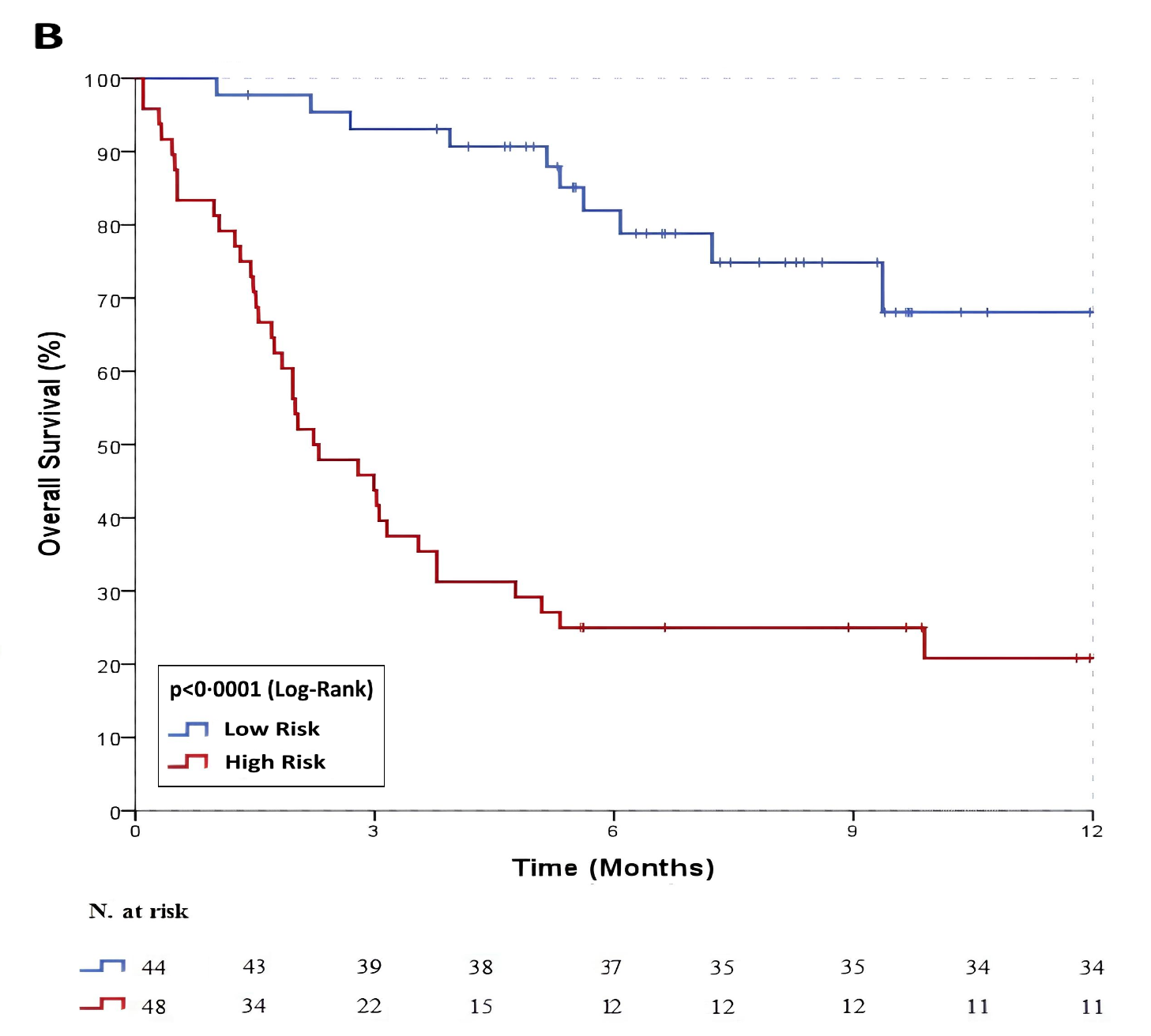


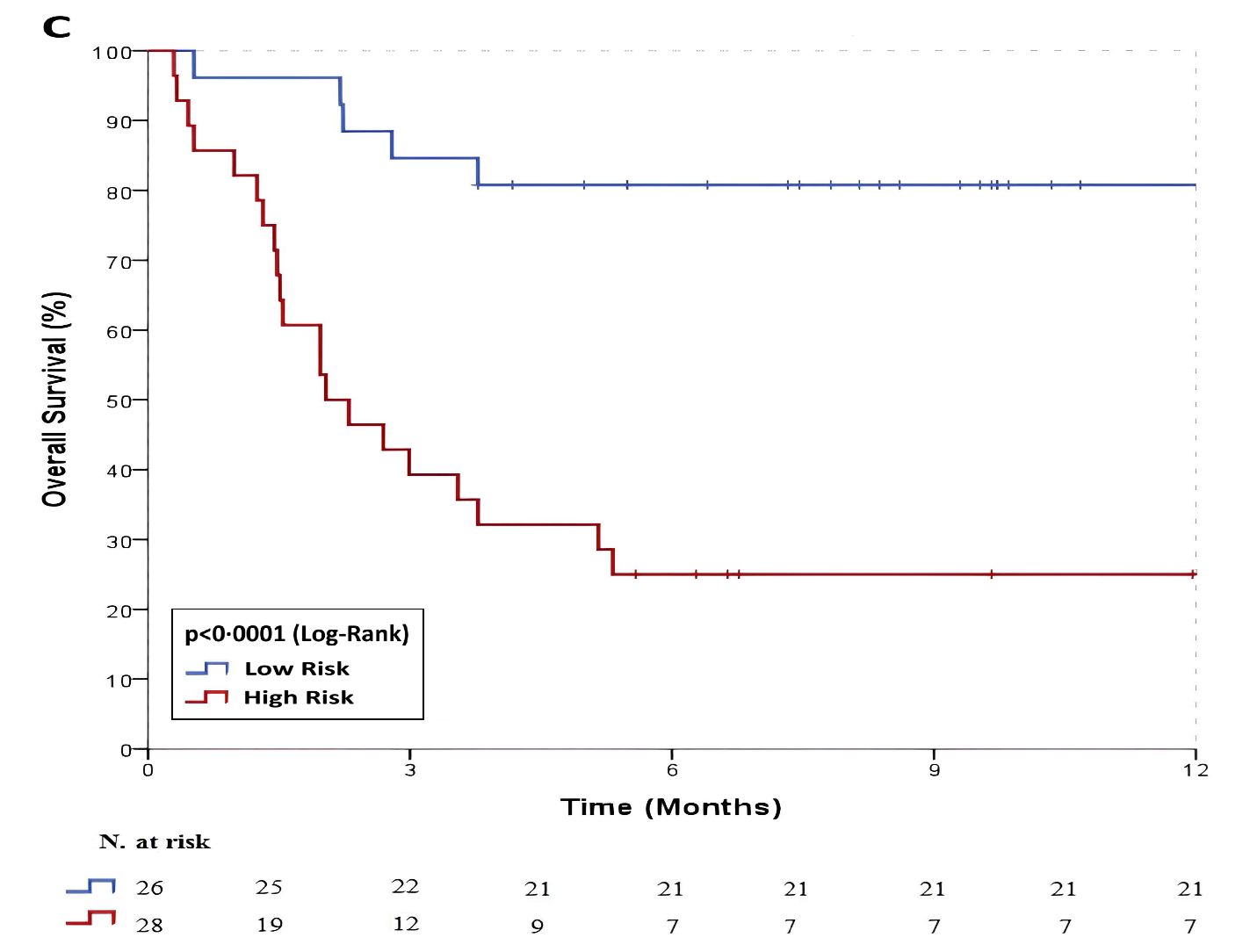


**Figure S5. Kaplan-Meier Survival Analysis in Breast Cancer Patients Stratified by Risk Scores**

(A) PROMISE score-based survival stratification (n=92) (B) GRIm score-based survival stratification (n=92) (C) CTI score-based survival stratification (n=54). The log-rank test was used to compare survival differences between risk groups.

**Abbreviations: CTI Score**: CRP-Triglyceride-Glucose Index, **GRIm Score**: Gustave Roussy Immune Score, **n:** sample size, **PROMISE Score**: Prognostic Score for Hospitalized Cancer Patients.


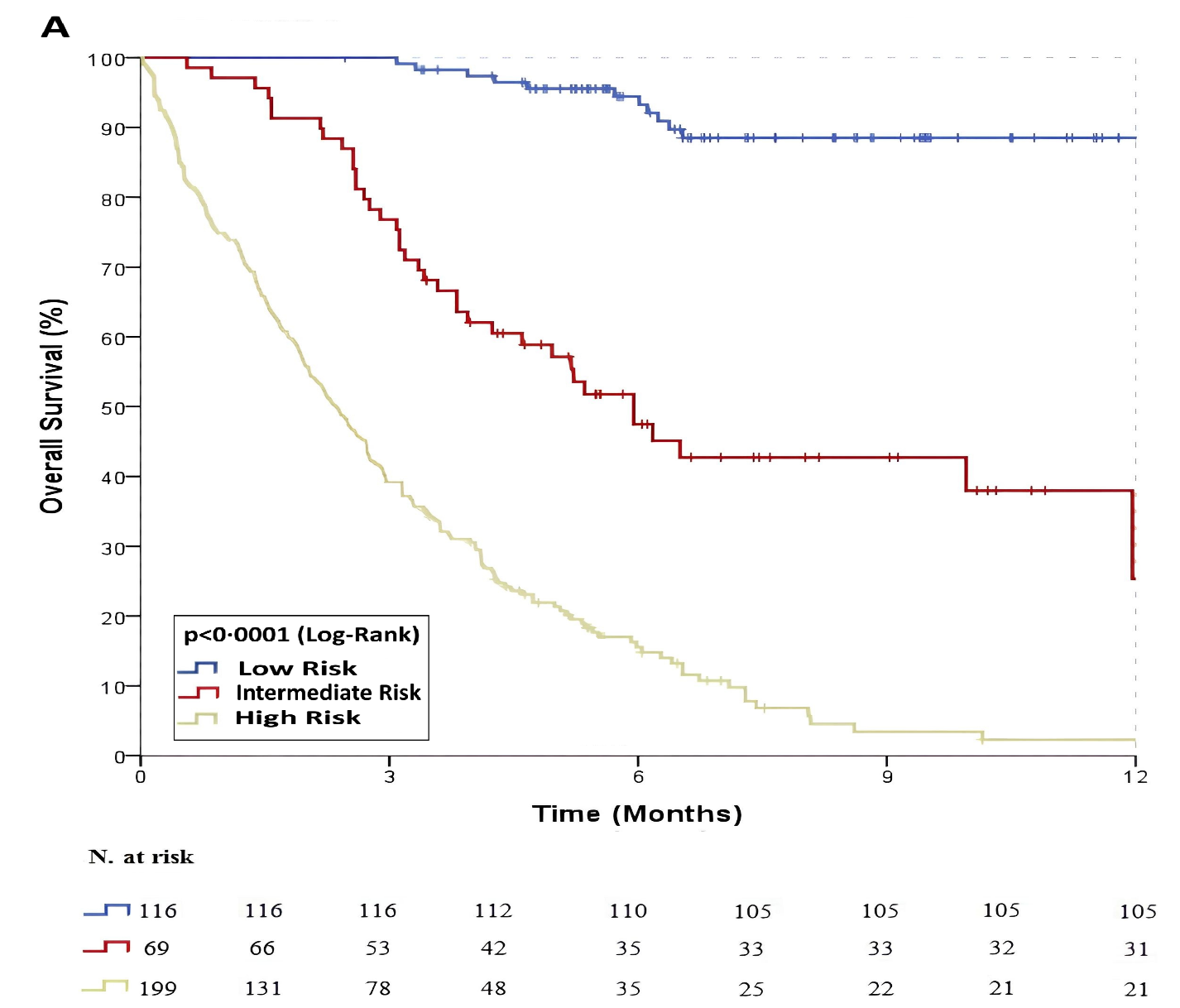


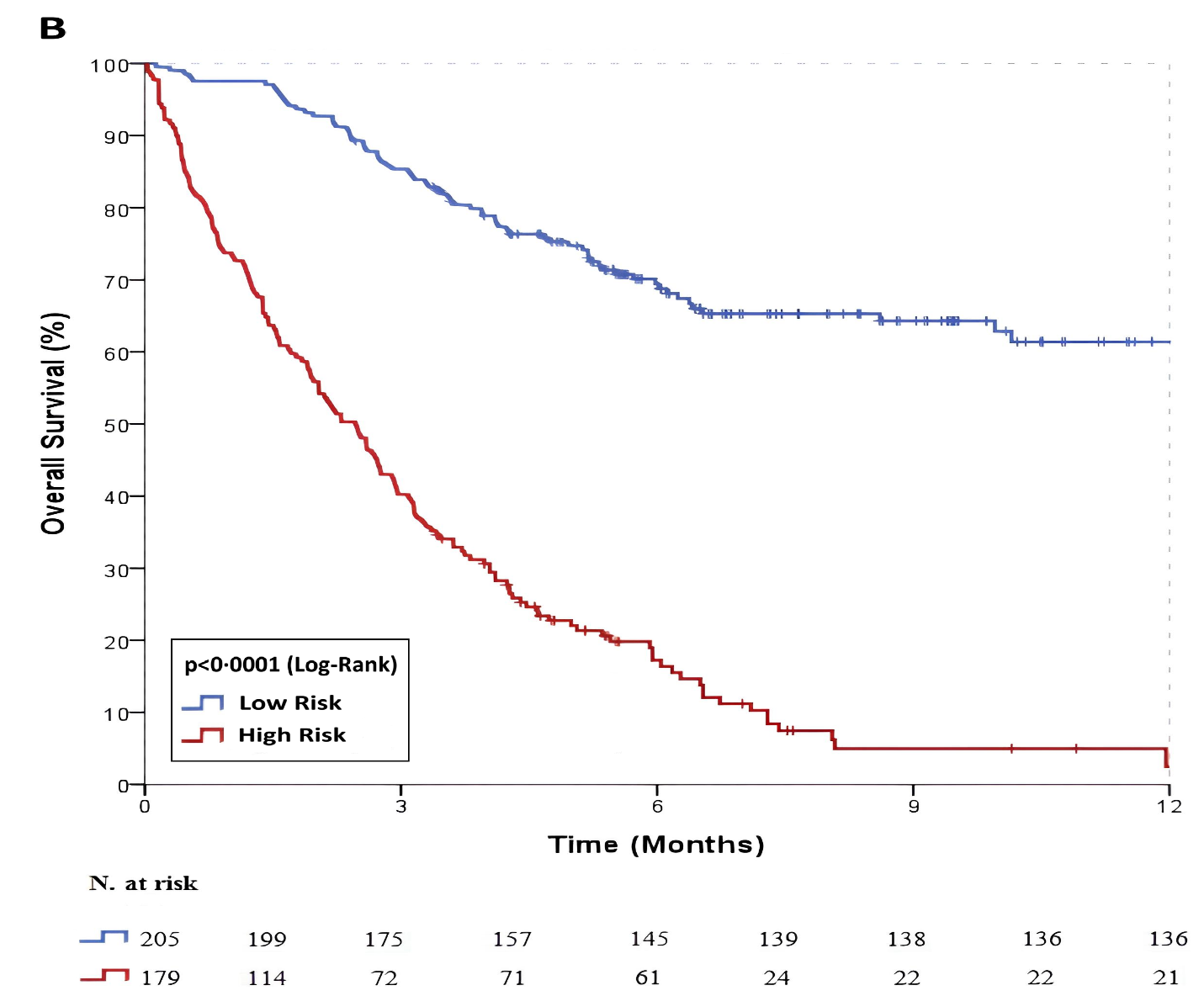


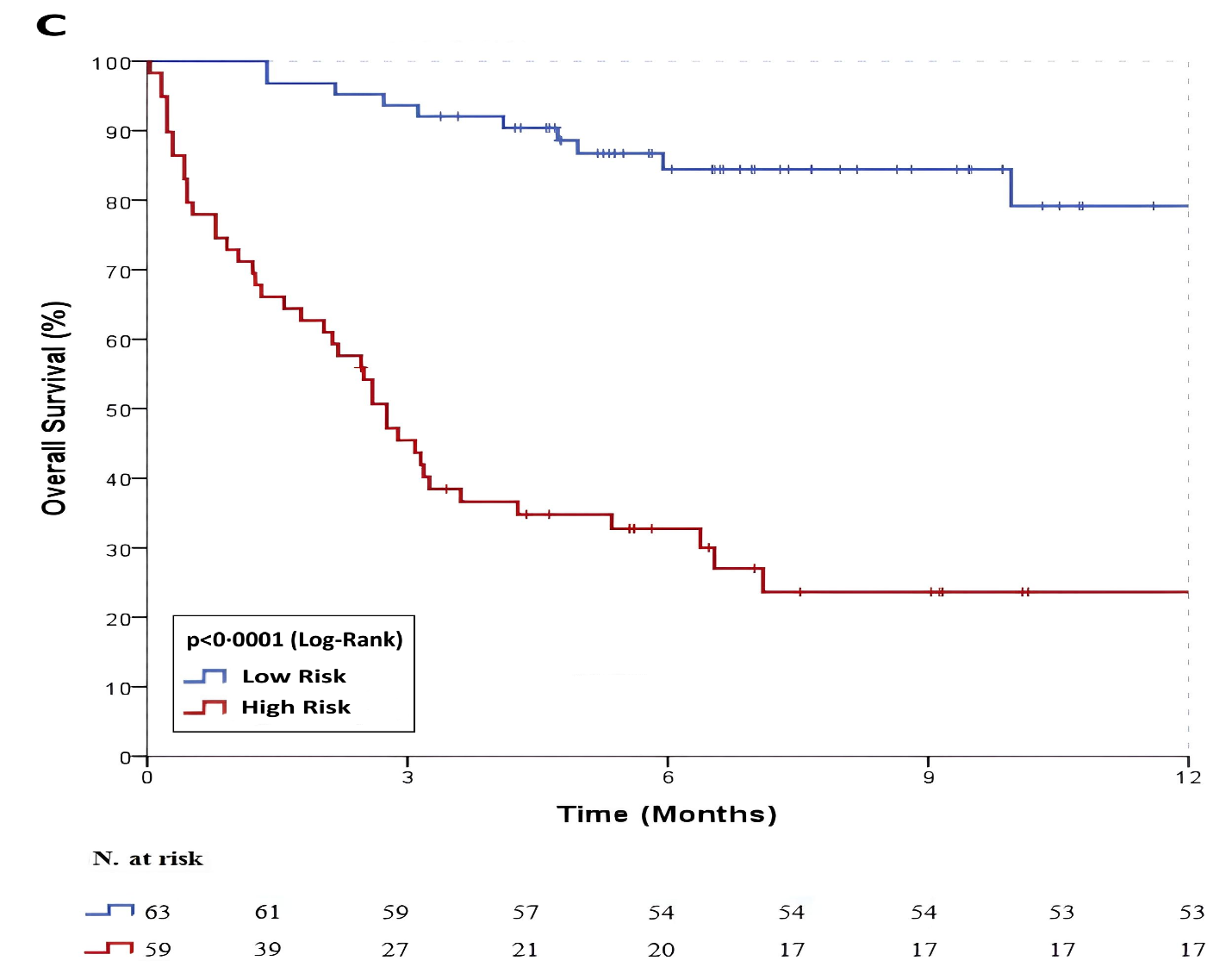


**Figure S6. Kaplan-Meier Survival Analysis in Patients with Other Cancers Stratified by Risk Scores**

(A) PROMISE score-based survival stratification (n=384) (B) GRIm score-based survival stratification (n=384) (C) CTI score-based survival stratification (n=122). The log-rank test was used to compare survival differences between risk groups.

**Abbreviations: CTI Score:** CRP-Triglyceride-Glucose Index, **GRIm Score:** Gustave Roussy Immune Score, **n:** sample size, **PROMISE Score:** Prognostic Score for Hospitalized Cancer Patients.

**Table S1. Baseline Clinical Characteristics of Patients According to CTI Score Availability**

| **Parameters (n=1109)** | **CTI Score Available, n=333 (%)** | **CTI Score Not Available, n=776 (%)** | **P values** |
| --- | --- | --- | --- |
| **Age** |  |  |  |
| <65 | 189 (56.8) | 406 (52.3) | p=0·17 |
| ≥65 | 144 (43.2) | 370 (47.7) |  |
| **Gender** |  |  |  |
| Female | 151 (45.3) | 308 (39.7) | p=0·08 |
| Male | 182 (54.7) | 468 (60.3) |  |
| **ECOG-PS** |  |  |  |
| 0-1 | 136 (40.8) | 250 (32.2) | p=0·07 |
| ≥2 | 197 (59.2) | 526 (67.8) |  |
| **Diagnosis** |  |  |  |
| GIS | 95 (28.5) | 255 (32.9) |  |
| Lung | 62 (18.6) | 221 (28.5) |  |
| Breast | 54 (16.2) | 38 (4.9) |  |
| Genitourinary | 29 (8.7) | 60 (7.7) | p= 0·19 |
| Gyn-oncology | 29 (8.7) | 55 (7.1) |  |
| Head-Neck | 12 (3.6) | 45 (5.8) |  |
| Brain | 16 (4.8) | 28 (3.6) |  |
| Others | 36 (10,9) | 74 (9.5) |  |
| **Treatment Response** |  |  |  |
| PD | 178 (53.5) | 551 (71.0) | p=0·07 |
| Non-PD | 155 (46.5) | 225 (29.0) |  |
| **Smoking Status** |  |  |  |
| Non-smoker | 91 (27.3) | 171 (22.0) |  |
| Former smoker | 201 (60.4) | 532 (68.6) | p=0·09 |
| Active smoker | 41 (12.3) | 73 (9.4) |  |
| **Dyslipidemia** |  |  |  |
| Absent | 255 (76.6) | 657 (84.7) | p=0·10 |
| Present | 78 (23.4) | 119 (15.3) |  |
| **Diabetes Diagnosis** |  |  |  |
| Absent | 237 (71.2) | 569 (73.3) | p=0·47 |
| Present | 96 (28.8) | 207 (26.7) |  |
| **Line of Systemic Therapy** |  |  |  |
| 1-2 | 288 (62.8) | 700 (90.2) | p=0·08 |
| ≥3 | 45 (37.2) | 76 (9.8) |  |
| **BMI (kg/m²)** |  |  |  |
| <22.5 | 117 (35.1) | 294 (37.9) | p=0·16 |
| ≥22.5 | 216 (64.9) | 482 (62.1) |  |
| **GRIm Score** |  |  |  |
| Low Risk | 170 (51.1) | 340 (43.8) | p=0·06 |
| High Risk | 163 (48.9) | 436 (56.2) |  |
| **PROMISE Score** |  |  |  |
| Low Risk | 114 (34.2) | 164 (21.1) |  |
| Intermediate Risk | 64 (19.2) | 151 (19.5) | p=0·10 |
| High Risk | 155 (46.6) | 461 (59.4) |  |
| **mCCI** |  |  |  |
| 6-7 | 74 (22.2) | 143 (18.4) | p=0·15 |
| ≥8 | 259 (77.8) | 633 (81.6) |  |
| **Albumin (g/dl)** |  |  |  |
| <35 | 224 (67.2) | 452 (58.2) | p=0·09 |
| ≥35 | 109 (32.8) | 324 (41.8) |  |
| **LDH (UI/L)** |  |  |  |
| <225 | 108 (32.4) | 318 (41.0) | p=0·11 |
| ≥225 | 225 (67.6) | 458 (59.0) |  |

**Abbreviations: BMI:** Body Mass Index, **CTI Score:** CRP-Triglyceride-Glucose Index, **ECOG-PS:** Eastern Cooperative Oncology Group Performance Status, **GRIm Score:** Gustave Roussy Immune Score; **LDH:** Lactate Dehydrogenase, **mCCI**: Modified Charlson Comorbidity Index, **non-PD:** Non-Progressive Disease, **PD:** Progressive Disease, **PROMISE Score:** Prognostic Score for Hospitalized Cancer Patients
